# Postbiotic Supplementation Increases Amino Acid Absorption from Plant-Based Meal: A Placebo-Controlled, Randomized, Double-Blind, Crossover Study

**DOI:** 10.1007/s12602-025-10480-y

**Published:** 2025-02-24

**Authors:** Christine M. Florez, Javier Zaragoza, Jessica Prather, Mandy Parra, Jaci Davis, Amie Vargas, Audrey Ross, Ralf Jäger, Martin Purpura, Simone Guglielmetti, Grant M. Tinsley, Lem Taylor

**Affiliations:** 1 Energy Balance & Body Composition Laboratory, Department of Kinesiology and Sport Management, Texas Tech University, Lubbock, TX USA; 2 Department of Health and Human Performance, Concordia University Chicago, River Forest, IL 60305 USA; 3 Human Performance Lab, School of Exercise Sport and Science, University of Mary Hardin-Baylor, Belton, TX USA; 4 Increnovo LLC, Whitefish Bay, WI USA; 5 μbEat Lab, Department of Biotechnology and Biosciences (BtBs), Università Degli Studi Di Milano-Bicocca, Milan, Italy; 6 Doctor of Physical Therapy Program, School of Health Professions, University of Mary Hardin-Baylor, Belton, TX USA

**Keywords:** Postbiotic, Plant protein, Amino acid absorption, γ-Irradiation

## Abstract

Supplementation of probiotic strains can enhance the absorption of amino acids from protein in the gut. The purpose of this study was to assess if supplementation of a multi-strain probiotic or a postbiotic, consisting of the same strains, would alter the absorption of individual and total amino acids following ingestion of a plant-based meal. Sixteen male participants consumed either probiotic (PRO) or postbiotic (cells inactivated by γ-irradiation; POST), both consisting of *L. paracasei* LP-DG® (CNCM I-1572) plus *L. paracasei* LPC-S01 (DSM 26760), or a placebo (PLA) for 2 weeks in a randomized, double-blind, crossover design study separated by a 4-week washout period. During the testing session, blood samples were taken at baseline, 30-, 60-, 120-, and 180-min post-ingestion of a plant-based vegan burger patty. Plasma amino acid levels were analyzed, and percent changes from baseline were assessed using linear mixed-effects models, with the PLA condition as the reference group. There was statistically significant POST condition-by-time interactions for percent changes in alanine, asparagine, citrulline, cystine, glycine, methionine, proline, and total amino acids (*p* < 0.05, for all). Additionally, there was a statistically significant condition (PRO) by time interactions for cystine (*p* = 0.02). Two weeks of POST supplementation resulted in significant improvements in amino acid absorption profiles for various individual amino acids and total amino acids compared to PLA. This is the first study to report improved amino acid absorption from a mixed macronutrient meal following a period of postbiotic supplementation.

**Supplementary Information:**

The online version contains supplementary material available at 10.1007/s12602-025-10480-y.

## Introduction

Probiotics are living microorganisms that can confer health benefits when administered in adequate amounts [1, 2]. Probiotic supplementation has been shown to elicit a myriad of benefits to the host; however, for a probiotic to impact health, it must survive the GI environment, and the magnitude of its effects is strain- and dose-dependent [3]. Moreover, many live microorganisms used as probiotics are sensitive to heat and/or oxygen, leading to challenges in sustaining their shelf-life. In fact, to offset the expected decrease in viable cells at the end of shelf-life, manufacturers often include more than the stated quantity [4]. Fermented foods such as kimchi, sauerkraut, yogurt, and kombucha also possess health-promoting bacteria; however, the number of bacteria in food products is not standardized. Furthermore, the bacteria in these foods, like probiotics, have limited shelf-life. For this reason, alternative methods of extending probiotic shelf-life have been explored, leading to the development of postbiotics [5, 6].

A broad consensus on the definition of postbiotics has not yet been established [7]. However, postbiotics must contain intact cells or cell fragments and are produced from killing bacteria through a deliberate killing process. They are “preparations of inanimate microorganisms and/or their components that confer a health benefit on the host.” [8]. These inactivated cells may offer an attractive alternative relative to safety [9] and stability, when compared to their probiotic counterparts [5]. The process of inactivation can be achieved in several ways, including thermal sterilization, pasteurization, or irradiation [5]. Regardless of their preparation method, postbiotics are thought to be potentially as effective as probiotic supplementation [10]. Of the purported benefits, certain strains of probiotics have been observed to support digestive health [11], positively modulate immune system function [12, 13], and aid in gastrointestinal protein absorption [14]. Research in probiotic and postbiotic supplementation continues to gain interest with an emerging focus on its potential to improve protein absorption efficiency.

Dietary protein intake is essential to basic human physiology and influences tissue-building, body composition, and immune function [15]. Plant proteins are typically considered to be low-quality when compared to animal sources due to their amino acid profiles [16]. However, despite this classification, the increased consumption of plant-based proteins is cited as a nutritional strategy to improve overall health [17]. Additionally, many individuals have opted to reduce meat consumption or avoid it altogether. A worldwide survey in 2019 revealed that 40% of those surveyed admitted to intentionally reducing meat consumption in favor of alternative protein sources [18]. In the USA, the plant-based meat market is anticipated to grow by several billion dollars [19]. Due to increased interest in plant-based diets, research to improve the bioavailability of plant-based proteins is currently being conducted [20]. One such strategy is the co-ingestion of bacterial cells alongside plant-based proteins to improve amino acid bioavailability.

*Lacticaseibacillus paracasei* is a species of bacteria frequently included in commercially available supplements [21]. Strains from this species have been previously reported to aid in improving the gut microbiota environment [22–24]. Additionally, we have previously demonstrated that *L. paracasei* DG and *L. paracasei* LPC-S01 increased amino acid absorption from pea protein powder when compared to a placebo [20]. Therefore, the purpose of this study was to assess if supplementation of a multi-strain probiotic or a postbiotic consisting of inactivated cells of the same strains would alter the absorption of individual and total amino acids following ingestion of plant-based meals. It was hypothesized that both probiotic and postbiotic supplementation would enhance amino acid appearance relative to placebo.

## Methods

### Probiotic and Postbiotic Preparations

Three microbial preparations were evaluated, each comprising a blend of industrially lyophilized biomasses from two *Lacticaseibacillus paracasei* strains: DG (L. casei DG®, CNCM I-1572) and LPC-S01 (DSM 26760). One preparation contained viable bacterial cells, another included bacteria inactivated by heat treatment (80 °C for 30 min), and the third consisted of bacteria inactivated by γ-irradiation (10 kGy, in accordance with ISO 11137). Only the viable (probiotic) and γ-irradiated (postbiotic) preparations were used in the human trial. The postbiotic, probiotic, and corresponding placebo (maltodextrin) were provided in sachets by Sofar S.p.A., Italy. The probiotic sachets contained 10 billion colony-forming units (CFU), while the postbiotic sachets contained 10 billion active fluorescent units (AFU), 5 billion of each individual strain, respectively.

### Evaluation of Membrane Integrity

The membrane integrity of bacteria was evaluated following labeling with SYTO™ 24 and propidium iodide using flow cytometry (BD Accuri™ C6 flow cytometer, BD Biosciences, Milan, Italy). One gram of lyophilizate was resuspended in PBS buffer to obtain a 1:10 dilution. Following homogenization, the cell suspension was subjected to serial decimal dilutions in filtered PBS buffer, using 1.5-mL Eppendorf tubes. These dilutions were used to determine vitality by setting the following instrumental parameters: sample acquisition volume: 50 µL and threshold: FSC: 4000 and SSC: 1000. The flow cytometric analysis involved an initial acquisition of the unlabeled sample to identify the appropriate decimal dilution for subsequent staining, ensuring that the number of events/microliter was within the range of 1000 to 3000. After determining the correct dilution, cell labeling was performed by adding 50 µL of the sample and 10 µL of the SYTO™ 24 marker to 440 µL of diluent (filtered PBS) (absorption (nm): DNA (490), RNA (ND)) along with propidium iodide (PI). The total number of viable cells was then determined by double labeling the samples in accordance with protocol B of the ISO 19344:2015 method (IDF 232).

### Evaluation of β-Galactosidase Activity

The β-galactosidase activity was assessed using a colorimetric test on the protein extract obtained from cell lysis of the freeze-dried final product. Specifically, 1 g of freeze-dried product was resuspended, washed twice in 20 mL of 0.1 M phosphate buffer (pH 7), and then resuspended in a final volume of 5 mL. Following cell disruption via bead beating (4 cycles of 20 s at 6300 RPM), the suspension was centrifuged at 13,000 RPM for 45 min at 5 °C. After protein quantification using the Bradford assay, a 100 μL aliquot of the protein extract was added to 130 μL of phosphate buffer and 100 μL of o-nitrophenyl-β-galactosidase (ONPG) (3 mg/mL) and incubated for 2 h at 37 °C, monitoring the change in absorbance at 420 nm. The enzymatic activity was expressed as a relative unit, calculated by comparing the enzymatic units of the sample with those of the “non-inactivated” sample of the respective product.

### Overview of Human Trial

A randomized, double-blind, crossover study was performed to assess the amino acid concentration in the blood after the administration of a plant-based burger containing 20 g of protein from pea protein isolates, rice protein, and mung bean protein with co-administration of probiotics or postbiotics vs. placebo. The subjects were asked to consume one sachet in the morning with 240 mL of water. The University of Mary Hardin-Baylor Institutional Review Board approved this study (IRB number: #114 on March 9, 2022). Participants were informed of the rationale and purpose of this study and their right to refuse or discontinue participation at any given time throughout the study. All participants provided written informed consent prior to participation.

### Participants

**Fig. 1:**
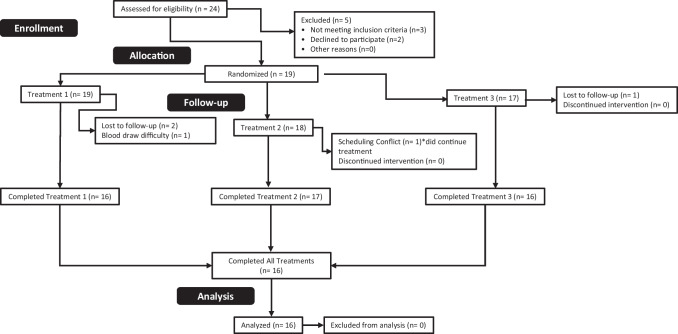
Consort chart

Sixteen normal-weight recreationally active males ([mean ± SD] age: 23.1 ± 3.2 years; height: 180.3 ± 7.7 cm; weight 86.6 ± 13.8 kg; body fat 17.8 ± 6.3%) were recruited for the present study. Subjects did not consume any nutritional or ergogenic supplement known to affect measures of the current study for 6 weeks prior to participation, including probiotics, prebiotics, and digestive enzymes. Exclusion criteria included any individual who was being treated for or diagnosed with a gastrointestinal, cardiac, respiratory, circulatory, musculoskeletal, metabolic, immune, autoimmune, psychiatric, hematological, neurological, or endocrinological disorder. Additionally, participants who were determined to not be weight stable, defined as measured body mass deviating by 2% or more between trials, and participants who were not willing to abstain from alcohol, nicotine, and caffeine for 12 h prior to each visit were excluded (Fig. 1).

### Study Design

**Fig. 2:**
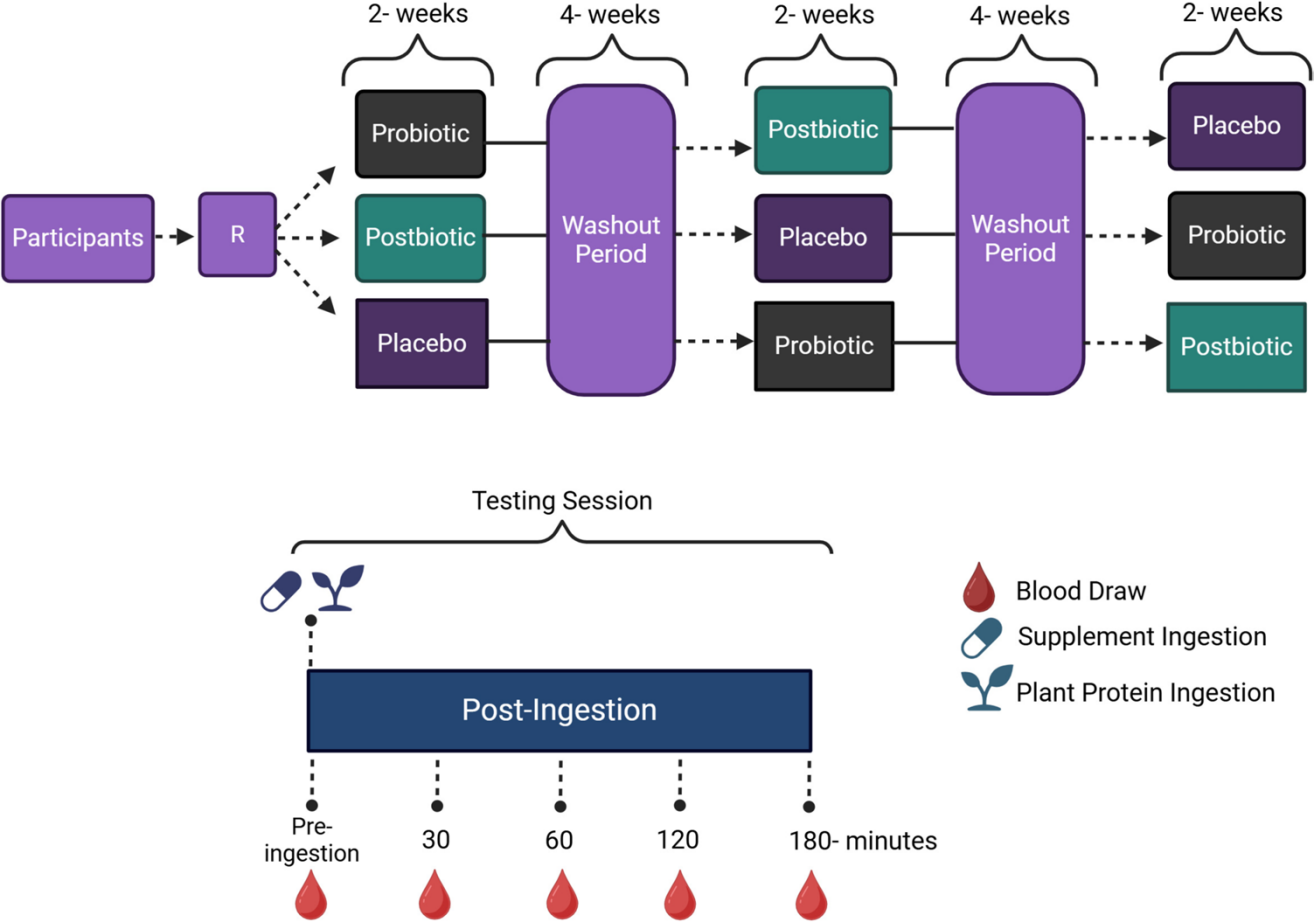
Study scheme for the intervention trial with the probiotic (PRO), postbiotic (POST), and placebo (PLA) preparations

Three supplementation periods that each spanned 2 weeks were completed and separated by a washout period of 4 weeks (Fig. 2). For each study visit, all participants reported to the laboratory between 06:00 and 9:00 h after an 8- to 10-h fast. Their diet was recorded, and subjects were asked to repeat the same diet for the 2 weeks leading up to the second and third experimental testing sessions.

Participants were randomly assigned to ingest either a placebo (PLA), a postbiotic (POST), or a probiotic (PRO) product for 2 weeks. On testing days, biotics were ingested fasted with 240 mL of water (or whatever the standard procedure was), while on non-testing days, the biotics were ingested upon waking with breakfast with 240 mL of water. Upon arrival for each study visit, participants had their resting heart rate, blood pressure, body mass, height, and body composition (InBody 770, InBody, Seoul, South Korea) measured.

### Testing Session

Subjects rested semi-supine for placement of a Teflon catheter into an antecubital vein for multiple blood sampling. The catheter was kept patent by flushing with 2–3 mL of 0.9% sodium chloride. Following baseline sampling, participants ingested their respective treatments and then consumed a vegan burger (Beyond Burger, Beyond Meat, El Segundo, CA). Thereafter, blood samples were collected at 30-, 60-, 120-, and 180-min post-ingestion (Fig. 2). Subsequently, a 4-week washout period was implemented, followed by the opposite condition.

### Vegan Burger Preparation

A single vegan patty (20 g protein, 14 g fat, and 7 g carbohydrate) was prepared in the lab on an indoor grill. The patty was cooked until the internal temperature reached 165° Fahrenheit as measured by a meat thermometer and served to participants plain.

### Outcome Variables

Dependent variables for the amino acid appearance portion of the trial included total amino acids, essential amino acids (EAAs), branched-chain amino acids (BCAAs), and individual amino acids (arginine, glutamine, citrulline, serine, asparagine, glycine, threonine, alanine, ornithine, methionine, proline, lysine, aspartic acid, histidine, valine, glutamic acid, tryptophan, leucine, phenylalanine, isoleucine, cysteine, and tyrosine) in peripheral blood. The maximum observed concentration (*C*_max_) and corresponding time (*T*_max_) and incremental area under the curve (iAUC) were calculated for each amino acid. Safety was assessed with a complete blood count (CBC) and comprehensive metabolic panel (CMP) at all pre- and post-time points.

### Amino Acid Analysis

Amino acid analysis was performed by Heartland Assays, Iowa State University Research Park, Ames, IA, USA. EZ:faast® amino acid analysis kits (Phenomenex, Torrance, CA) were used for liquid chromatographic analysis of amino acids using tandem-mass spectrometry (LC/MS/MS) and electrospray ionization (ESI). The procedure consisted of solid phase extraction of 25 µL of plasma with internal standards by a sorbent tip attached to a syringe with an eluting solvent (a 3:2 mixture of sodium hydroxide with 77% n-propanol and 23% 3-picoline). The free amino acids were then derivatized by adding a mixture of 17.4% propyl chloroformate, 11% isooctane, and 71.6% chloroform. The resulting mixture was vortexed and allowed to sit at room temperature for 1 min, followed by liquid–liquid extraction with isooctane. Fifty microliters of the organic layer was removed, dried under nitrogen gas, and suspended in the HPLC run solvents before being injected into the LC/MS/MS. Chromatographic separation of the derivatized amino acids was conducted on an EZ:faast amino acid analysis-mass spectrometry column (250 × 2.0 mm i.d., 4 µm) using an Agilent 6460 triple quadrupole LC/MS/MS system (Santa Clara, CA). Ten millimolar of ammonium formate in water with 0.2% formic acid (mobile phase A) and 10 mM ammonium formate in methanol with 0.2% formic acid (mobile phase B) were used as a solvent system with gradient conditions of 68% B at 0 min to 83% B over 13 min with a flow rate of 0.25 mL/min. Amino acids and internal standard data were collected using the Dynamic Multiple Reaction Monitoring mode using Mass Hunter acquisition software (Agilent, Santa Clara, CA). Mass Hunter Quantitation software was used to quantitate the unknown plasma samples based on best-fit standard curves [20].

### Statistical Analysis

Seventeen individuals participated in the study. One participant was missing all data from all time points within one condition and was therefore excluded from the analysis. For the remaining 16 participants, the only missing data point was the 180-min time point for one participant within the placebo condition. These missing data were imputed using the last observation carried forward from the 120-min time point. Therefore, 16 individuals were included in the statistical analysis.

Changes in raw amino acid concentrations and percent changes from baseline were analyzed using linear mixed-effects models (nlme package [25], v. 3.1–157) with a random intercept for the participant. A first-order autoregressive (AR1) variance–covariance matrix was employed, using the correlation form of time | participant/condition. These models were fit by maximizing the restricted log-likelihood (REML). In all models, the reference condition was placebo (PLA). Model assumptions were examined through graphical methods (i.e., residuals vs. fitted plots and quantile–quantile plots). Model coefficients were examined using the SjPlot package [26] (v. 2.8.14) to determine the effects of each condition, time, and condition-by-time interactions.

In addition to linear mixed-effects models, pharmacokinetic calculations were performed. The incremental area under the concentration vs. time curve (iAUC) was calculated using the method of Brouns et al. [27]. The PKNCA package [28] (v. 0.10.0) was used to establish the maximum observed concentration (*C*_max_) and time of maximum observed concentration (*T*_max_). iAUC, *C*_max_, and *T*_max_ values were analyzed via the Friedman rank sum test, using the rstatix package [29] (v. 0.7.0). When a Friedman test was significant, post hoc tests were performed using Wilcoxon signed-rank tests. For all tests, statistical significance was accepted at *p* < 0.05. Data were analyzed in R (version 4.2.1).

## Results

### Selection of the Postbiotic Formulation

**Fig. 3:**
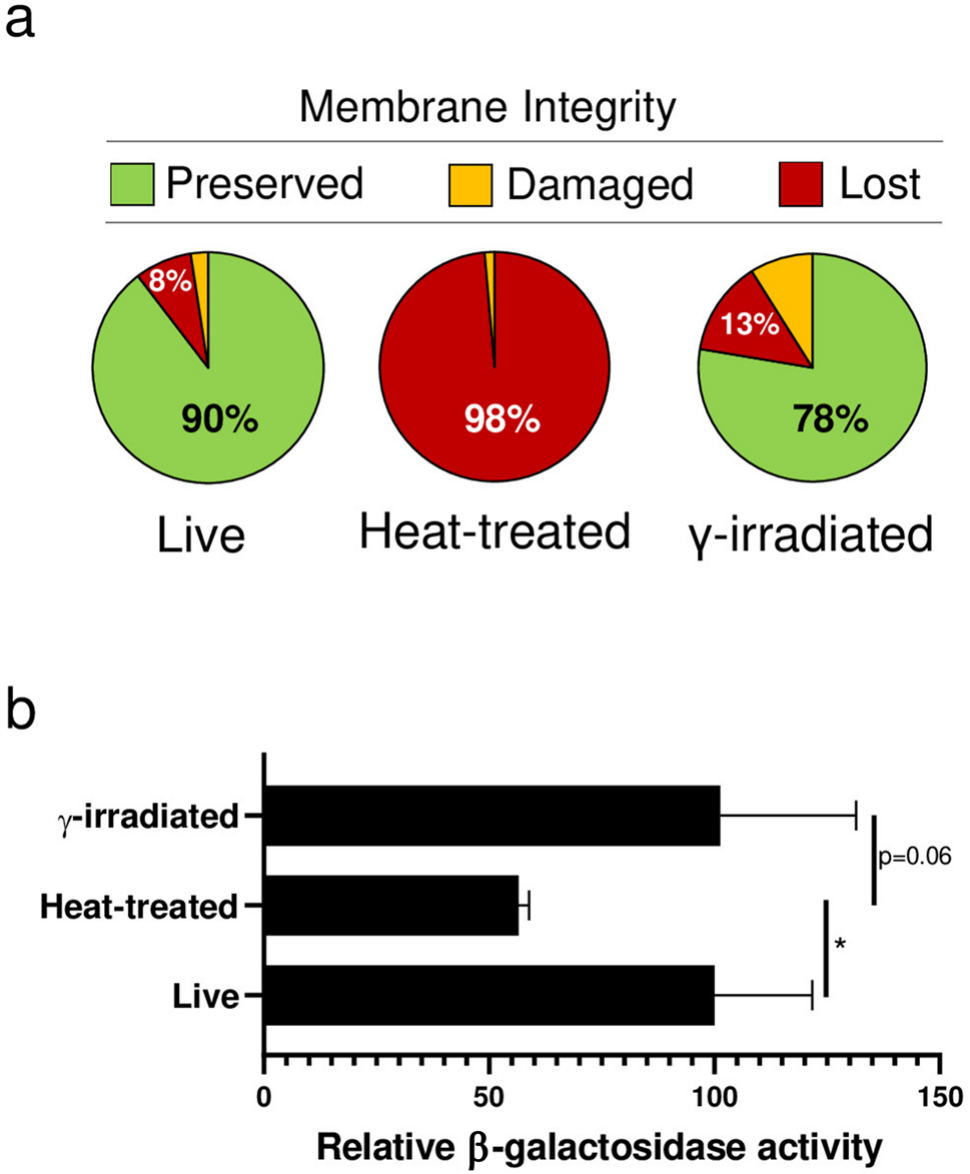
Comparison of lyophilized biomasses of *Lacticaseibacillus paracasei* LPC-S01 before (live) and after inactivation by heat treatment or γ-irradiation. **a** Cell membrane integrity assessed by flow cytometry following SYTO-9/propidium iodide staining, with data presented as the percentage of fluorescent units in the 2D pie chart. **b** β-Galactosidase activity assessed through the ONPG (ortho-nitrophenyl-β-galactoside) hydrolysis assay; statistical analysis was performed using an unpaired Student’s *t*-test (*n* = 3; *p < 0.05)

The industrial bacterial biomass of the strain *L. paracasei* LPC-S01 was used to determine the most suitable method for producing the postbiotic for the subsequent human study. To this end, we compared the live, heat-inactivated, and γ-irradiated preparations in terms of cell membrane permeability to propidium iodide and intracellular enzymatic activity (β-galactosidase). Both heat treatment and γ-radiation completely inactivated the *L. paracasei* LPC-S01 biomass (CFU per gram of powder below the detection limit). Nonetheless, we found that ionizing radiation (γ rays) inactivated bacterial cells while preserving membrane integrity to a much greater extent than heat treatment (Fig. 3a). Additionally, the β-galactosidase activity in γ-inactivated cells was not significantly lower than that in viable cells, whereas in heat-inactivated bacteria, it was significantly reduced (Fig. 3b). Based on these findings, bacterial cells inactivated by γ-irradiation were selected as the postbiotic for the human trial, to be compared with the same live probiotic strains.

### Amino Acid Concentrations

**Fig. 4:**
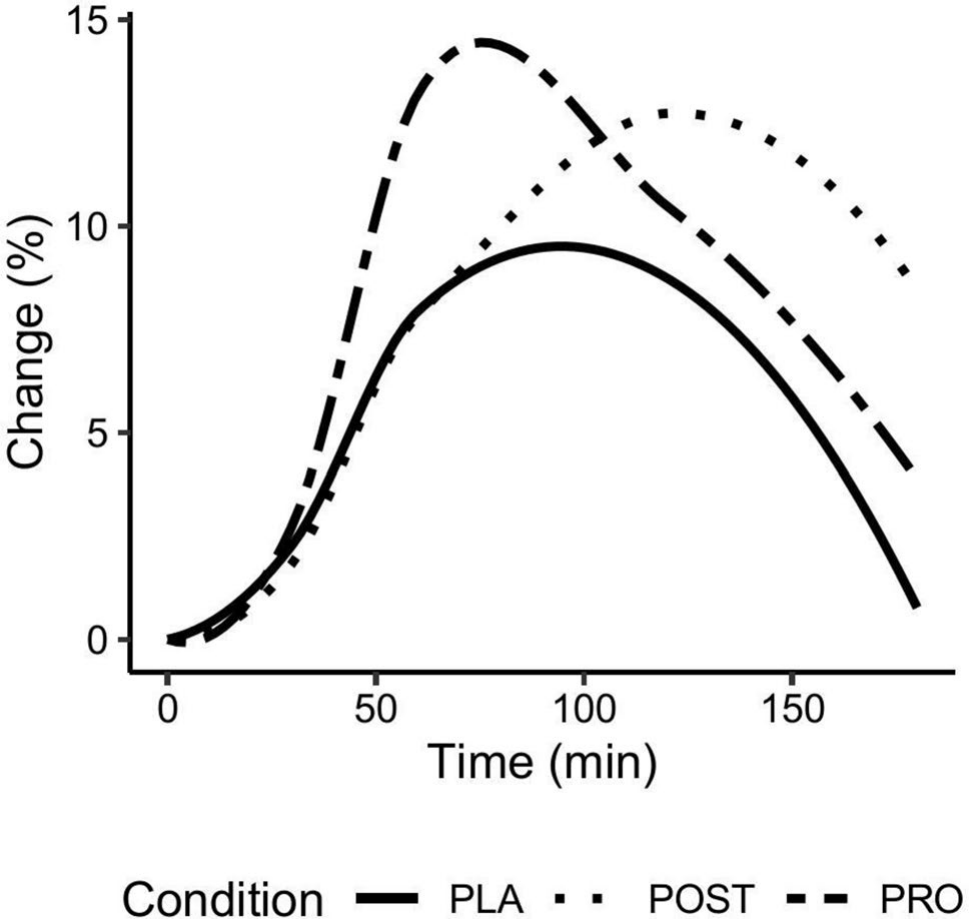
Percent change in total amino acids. Percent changes in total amino acids following postbiotic (POST), probiotic (PRO), or placebo (PLA) are displayed. In the linear mixed-effects model, a statistically significant group by time interaction term was present for POST vs. PLA (*p* = 0.04), but not PRO vs. PLA (*p* = 0.56)

Relative to the reference model (i.e., PLA condition), there were statistically significant POST condition-by-time interactions for percent changes in alanine (0.07%/min [0.01–0.13]; *p* = 0.02), asparagine (0.08%/min [0.01–0.15]; *p* = 0.03), citrulline (0.06%/min [0.01–0.12]; *p* = 0.02), cystine (0.04%/min [0.01–0.08]; *p* = 0.02), glycine (0.08%/min [0.02–0.14]; *p* = 0.01), methionine (0.05%/min [0.01–0.10]; *p* = 0.02), proline (0.05%/min [0.0–0.10]; *p* = 0.04), and total amino acids (0.05%/min [0.0–0.09]; *p* = 0.04; Fig. 4, Table 1). Additionally, there was a statistically significant PRO condition-by-time interactions for cystine (0.05%/min [0.01–0.08]; *p* = 0.02). In addition to these condition-by-time interactions, significant effects of time were observed for alanine (*p* = 0.02), arginine (*p* = 0.02), BCAAs (*p* < 0.001), citrulline (*p* = 0.01), cystine (*p* < 0.001), EAA (*p* = 0.03), isoleucine (*p* < 0.001), leucine (*p* < 0.001), methionine (*p* < 0.001), ornithine (*p* < 0.001), and valine (*p* = 0.002). Percent changes in amino acid concentrations over time are displayed in Fig. 4 and Table 1. Raw amino acid concentrations and coefficients and *p*-values for percent change linear mixed-effects models are displayed in the supplementary materials.

**Table 1:** Changes in amino acid concentrations

Amino acid	Condition	Mean percent changes (%)				Condition by time interaction vs. PLA
		0–30 min	0–60 min	0–120 min	0–180 min	*p*-value
Alanine	PLA	− 2.1	1.2	1.5	− 7.7	–
	POST	− 3.0	2.9	9.3	3.7	0.02*
	PRO	1.6	11.5	5.1	− 3.9	0.79
Arginine	PLA	21.3	37.4	42.1	18.4	–
	POST	29.1	43.4	46.2	27.5	0.60
	PRO	27.7	48.8	41.4	22.5	0.92
Asparagine	PLA	15.3	19.8	16.1	9.5	–
	POST	4.2	17.0	23.4	16.6	0.03*
	PRO	11.1	27.6	23.8	10.7	0.48
Aspartic acid	PLA	18.6	28.7	28.8	18.4	–
	POST	30.7	18.8	32.0	45.1	0.67
	PRO	27.6	102.8	11.1	14.4	0.61
Bcaa	PLA	3.3	13.4	19.6	10.7	–
	POST	2.5	10.6	18.6	16.1	0.29
	PRO	4.6	17.8	21.6	13.2	0.73
Citrulline	PLA	4.1	1.1	− 5.3	− 5.3	–
	POST	3.7	0.4	− 0.3	5.6	0.02*
	PRO	2.0	2.9	1.6	− 1.5	0.18
Cystine	PLA	− 1.4	− 6.6	− 10.5	− 12.5	–
	POST	− 5.6	− 6.4	− 2.0	− 8.9	0.02*
	PRO	− 4.4	− 5.8	− 7.7	− 5.4	0.02*
Eaa	PLA	3.8	12.1	14.6	6.0	–
	POST	2.9	10.6	17.0	12.1	0.13
	PRO	5.0	17.7	17.9	8.5	0.65
Glutamic acid	PLA	12.0	14.8	8.6	− 1.2	–
	POST	17.9	24.3	22.1	25.8	0.07
	PRO	7.5	19.3	11.9	− 3.0	0.96
Glutamine	PLA	− 3.1	1.0	2.1	− 2.7	–
	POST	− 1.2	2.2	6.0	4.0	0.16
	PRO	− 2.7	4.4	2.7	2.0	0.42
Glycine	PLA	1.9	3.5	2.1	− 4.2	–
	POST	− 0.4	2.9	9.0	7.7	0.01*
	PRO	2.2	12.5	5.3	0.2	0.51
Histidine	PLA	1.1	2.3	3.5	0.9	–
	POST	− 1.4	3.8	10.6	6.0	0.08
	PRO	1.4	9.1	6.9	1.7	0.88
Isoleucine	PLA	8.2	24.5	33.8	19.2	–
	POST	6.0	20.1	34.7	29.2	0.17
	PRO	9.4	29.3	39.0	25.5	0.42
Leucine	PLA	3.4	17.2	23.3	12.7	–
	POST	5.6	16.4	21.4	21.1	0.33
	PRO	6.8	23.8	26.7	15.8	0.81
Lysine	PLA	9.6	22.5	21.1	9.9	–
	POST	8.9	20.3	28.8	17.1	0.15
	PRO	13.0	31.4	29.9	13.7	0.58
Methionine	PLA	− 2.9	− 0.6	− 5.8	− 14.0	–
	POST	− 2.3	0.6	1.5	− 5.7	0.02*
	PRO	− 1.6	5.4	0.6	− 7.5	0.12
Ornithine	PLA	21.7	38.2	48.7	37.3	–
	POST	15.5	31.2	48.0	47.4	0.21
	PRO	24.3	43.5	45.5	38.5	0.84
Phenylalanine	PLA	3.4	10.8	14.0	2.7	–
	POST	3.8	12.2	16.5	10.8	0.11
	PRO	6.0	18.8	16.9	7.3	0.57
Proline	PLA	1.9	5.9	5.0	− 5.3	–
	POST	4.1	8.1	7.2	5.7	0.04*
	PRO	3.3	12.5	6.2	− 0.5	0.49
Serine	PLA	11.6	12.1	11.0	1.6	–
	POST	3.7	8.0	13.0	11.2	0.07
	PRO	10.3	19.4	13.2	10.3	0.28
Threonine	PLA	3.5	7.8	6.4	0.4	–
	POST	1.5	5.3	9.2	6.3	0.10
	PRO	3.5	15.0	11.7	4.5	0.33
Total AA	PLA	2.3	7.9	8.7	0.8	–
	POST	1.9	7.8	12.7	8.5	0.04*
	PRO	2.8	13.2	10.5	3.9	0.56
Tryptophan	PLA	1.1	3.9	2.6	− 7.7	–
	POST	0.7	9.7	5.8	1.5	0.12
	PRO	− 1.9	4.9	− 1.2	− 7.4	0.99
Tyrosine	PLA	1.0	15.1	11.6	1.2	–
	POST	− 1.1	16.1	12.9	7.7	0.33
	PRO	1.7	13.7	14.0	6.3	0.46
Valine	PLA	1.8	8.0	13.2	6.9	–
	POST	0.0	4.8	12.6	9.8	0.37
	PRO	2.0	11.4	13.8	8.3	0.83

*PLA* placebo, *POST* postbiotic, *PRO* probiotic

*Denotes significance

When examining raw concentrations without accounting for baseline differences, the only statistically significant condition-by-time effects was for higher citrulline (0.02 μmol/L/min [0.0–0.04]; *p* = 0.04) and cystine (0.03 μmol/L/min [0.0–0.06]; *p* = 0.04) over time in the POST condition relative to the reference model. In addition to these condition-by-time interactions, significant effects of time were observed for arginine (*p* = 0.04), BCAA (*p* = 0.002), citrulline (*p* = 0.03), cystine (*p* < 0.001), EAA (*p* = 0.048), isoleucine (*p* < 0.001), leucine (*p* = 0.001), methionine (*p* = 0.01), ornithine (*p* < 0.001), and valine (*p* = 0.03). Coefficients and *p*-values for raw concentration linear mixed-effects models are displayed in the supplementary material.

### Pharmacokinetic Analysis

Arginine iAUC values significantly differed by condition (*p* = 0.0498), with post hoc tests indicating higher iAUC in the POST condition as compared to PLA (*p* = 0.04; Table 2). Additionally, trends (0.05 < *p* < 0.1) were present for alanine (*p* = 0.07) and phenylalanine (*p* = 0.099) iAUC values. For *C*_max_, a significant Friedman test result was observed only for alanine (*p* = 0.047). However, post hoc tests did not reveal any significant differences between individual conditions (Table 3). For *T*_max_, significant effects of condition were observed for isoleucine (*p* = 0.03), methionine (*p* = 0.04), ornithine (*p* = 0.03), and total amino acids (*p* = 0.04). For isoleucine, post hoc tests indicated a significant difference between POST and PLA (*p* = 0.01), such that *T*_max_ tended to occur later in POST as compared to PLA (Table 4). For methionine, post hoc tests did not reveal any significant differences between individual conditions. For ornithine, post hoc tests indicated a significant difference between POST and PRO (*p* = 0.02), such that *T*_max_ tended to occur later in POST as compared to PRO. For total amino acids, post hoc tests revealed a significant difference between POST and PRO (*p* = 0.03), such that *T*_max_ tended to occur later in POST as compared to PRO. Additionally, trends for significant Friedman tests were observed for phenylalanine (*p* = 0.08), proline (*p* = 0.06), and threonine (*p* = 0.09).

**Table 2:** Incremental area under the curve (iAUC)

Amino acid	Postbiotic				Probiotic				Placebo				*p* (Friedman test)	Mean percent differences		Median percent differences	
	Mean	SD	Median	IQR	Mean	SD	Median	IQR	Mean	SD	Median	IQR		POST vs. PLA	PRO vs. PLA	POST vs. PLA	PRO vs. PLA
Alanine	2129	3504	883	2220	2474	2722	1416	2394	1418	1569	907	2237	0.07	40.1	54.3	− 2.7	43.8
Arginine	2796	942	2745.5*	1313	2654	1003	2482	1477	2323	1003	2345	1652	0.0498*	18.5	13.3	15.7	5.7
Asparagine	683	406	697	609	799	311	764	472	691	380	632	327	0.65	− 1.3	14.5	9.7	18.9
Aspartic acid	135	175	62	129	195	370	78	101	104	109	71	148	0.65	25.7	60.9	− 14.4	9.5
Bcaa	5193	2625	5056	2861	5923	3549	4902	5353	5441	3293	5406	3949	0.83	− 4.7	8.5	− 6.7	− 9.8
Citrulline	171	212	51	316	151	167	100	258	128	141	105	214	0.36	28.8	16.9	− 69.8	− 5.4
Cystine	207	784	0	14	62	130	0	35	36	92	0	4	0.98	141.0	53.6	NA	NA
Eaa	10,197	5778	11,059	8895	11,158	6711	8664	9799	9813	5425	9271	8346	0.78	3.8	12.8	17.6	− 6.8
Glutamic acid	655	498	552	617	577	523	472	779	449	519	316	821	0.28	37.5	25.0	54.4	39.5
Glutamine	2920	2348	2933	3044	2959	2962	2350	4748	2052	1981	1685	3562	0.61	34.9	36.2	54.0	33.0
Glycine	1325	1572	785	1451	1653	2016	943	1875	921	746	949	604	0.65	36.0	56.9	− 18.9	− 0.7
Histidine	538	408	556	664	483	446	420	267	347	372	207	441	0.14	43.3	32.8	91.5	67.9
Isoleucine	1468	743	1390	514	1653	876	1442	1436	1515	862	1463	884	0.44	− 3.2	8.7	− 5.1	− 1.5
Leucine	2030	1009	2007	1443	2194	1197	1795	1571	1967	1147	1849	1698	0.94	3.1	10.9	8.2	− 2.9
Lysine	3118	2127	3046	2098	3213	1556	2685	2266	3001	2508	2349	1977	0.37	3.8	6.8	25.9	13.4
Methionine	105	115	68	188	105	113	84	169	67	90	28	99	0.82	43.8	44.0	83.3	99.3
Ornithine	1437	653	1509	764	1400	677	1221	902	1442	605	1469	753	0.94	− 0.4	− 3.0	2.7	− 18.5
Phenylalanine	641	370	639	490	683	429	537	688	555	343	451	380	0.10	14.4	20.7	34.6	17.5
Proline	1124	928	944	1210	1155	1033	951	1539	919	871	621	1131	0.74	20.1	22.8	41.3	42.1
Serine	861	670	803	637	1119	988	912	1363	826	524	744	705	1.00	4.2	30.2	7.5	20.3
Threonine	863	648	751	1051	1095	845	913	1389	782	567	667	415	0.37	9.8	33.3	11.8	31.2
Total AA	22,228	14,118	20,381	19,566	21,384	14,899	14,492	20,628	18,647	12,116	15,148	14,431	0.47	17.5	13.7	29.5	− 4.4
Tryptophan	381	433	289	509	197	240	140	232	322	501	114	435	0.59	16.9	− 48.4	87.2	20.9
Tyrosine	607	735	517	478	627	455	604	603	672	757	487	494	0.83	− 10.2	− 7.0	6.0	21.5
Valine	1791	1084	1636	1425	2104	1530	1603	2441	2036	1303	2099	1623	0.72	− 12.8	3.3	− 24.8	− 26.8

*POST* postbiotic, *PRO* probiotic, *PLA* placebo

*Denotes significance

**Table 3:** Maximal observed concentrations (*C*_max_)

Amino acid	Postbiotic				Probiotic				Placebo				*p* (Friedman test)	Mean percent differences		Median percent differences	
	Mean	SD	Median	IQR	Mean	SD	Median	IQR	Mean	SD	Median	IQR		POST vs. PLA	PRO vs. PLA	POST vs. PLA	PRO vs. PLA
Alanine	375.9	98.5	335.4	125.6	372.7	77.6	368.2	138.3	361.3	90.4	362.5	146.0	0.047*	4.0	3.1	− 7.8	1.6
Arginine	122.0	24.9	121.3	33.2	124.5	19.1	122.6	23.5	125.4	24.5	124.6	34.2	0.65	− 2.7	− 0.7	− 2.7	− 1.6
Asparagine	58.1	11.5	59.6	15.7	59.8	8.1	59.5	8.3	59.0	11.0	55.4	16.2	0.47	− 1.6	1.2	7.3	7.2
Aspartic acid	7.9	3.2	7.5	2.9	10.7	10.9	7.3	2.8	7.2	3.7	6.7	3.8	0.44	9.6	39.4	10.2	7.7
Bcaa	548.3	96.9	537.4	66.4	563.9	115.5	523.4	170.0	575.2	124.4	545.9	97.9	0.83	− 4.8	− 2.0	− 1.6	− 4.2
Citrulline	33.9	7.1	34.4	9.8	32.9	6.7	31.7	5.7	32.1	6.2	31.9	6.8	0.17	5.5	2.3	7.6	− 0.6
Cystine	72.3	26.2	61.8	27.3	72.9	16.8	71.4	28.9	71.0	18.9	66.7	20.4	0.30	1.7	2.6	− 7.7	6.8
Eaa	1157.6	225.5	1111.7	237.3	1168.6	205.8	1132.6	283.5	1190.0	271.8	1151.5	207.5	0.83	− 2.8	− 1.8	− 3.5	− 1.7
Glutamic acid	48.1	15.6	49.4	22.4	51.6	18.8	47.2	33.3	45.7	19.7	38.2	24.9	0.27	5.1	12.0	25.6	21.1
Glutamine	619.4	88.6	631.0	133.7	646.6	162.6	614.5	70.1	618.7	84.5	607.8	102.1	0.57	0.1	4.4	3.8	1.1
Glycine	244.1	68.8	245.4	55.5	252.7	53.6	246.2	72.3	242.3	64.7	236.5	71.7	0.27	0.7	4.2	3.7	4.0
Histidine	88.4	12.9	86.8	22.5	87.1	12.4	84.6	15.9	91.1	19.3	85.9	21.6	0.94	− 3.0	− 4.5	1.0	− 1.5
Isoleucine	99.4	18.6	98.5	20.7	102.7	18.6	99.4	19.8	105.6	24.2	102.5	27.0	0.65	− 6.0	− 2.8	− 4.0	− 3.1
Leucine	172.1	34.1	166.4	28.0	176.4	35.5	164.5	57.3	181.3	40.3	175.0	44.5	0.30	− 5.2	− 2.7	− 5.0	− 6.2
Lysine	219.3	77.1	195.9	43.0	218.3	49.4	211.3	49.5	229.2	86.4	209.0	44.9	0.37	− 4.4	− 4.9	− 6.5	1.1
Methionine	31.0	7.5	30.0	9.9	30.4	5.9	30.5	6.9	31.7	7.7	31.3	9.6	0.57	− 2.4	− 4.5	− 4.2	− 2.6
Ornithine	69.4	16.5	61.8	21.1	68.4	12.5	63.7	21.2	70.2	19.0	61.5	29.6	0.83	− 1.2	− 2.6	0.5	3.5
Phenylalanine	71.6	10.9	69.3	13.8	71.6	10.5	73.4	13.6	75.5	13.2	74.5	18.5	0.47	− 5.4	− 5.3	− 7.2	− 1.5
Proline	201.0	55.1	203.9	49.5	198.2	55.9	199.3	50.5	203.7	77.5	181.4	35.9	0.65	− 1.3	− 2.7	11.7	9.4
Serine	104.6	26.3	97.6	38.9	110.3	21.1	112.0	28.7	105.5	26.3	101.7	29.2	0.30	− 0.8	4.5	− 4.1	9.6
Threonine	138.5	35.5	133.5	59.2	139.5	28.4	133.3	28.0	136.1	40.0	131.1	45.1	0.83	1.8	2.5	1.8	1.7
Total AA	3127.4	555.2	3141.5	695.1	3162.9	440.9	3189.4	697.1	3162.8	636.6	3087.1	511.5	0.47	− 1.1	0.0	1.7	3.3
Tryptophan	66.6	12.6	61.9	20.2	63.7	11.2	62.0	11.3	66.0	18.9	59.9	8.7	0.83	0.9	− 3.5	3.2	3.3
Tyrosine	80.8	24.5	73.5	27.3	78.1	13.8	75.6	19.0	84.2	33.7	79.7	24.5	0.57	− 4.1	− 7.5	− 8.1	− 5.4
Valine	278.9	46.8	273.4	37.5	288.2	69.4	265.8	77.6	291.2	66.7	279.8	48.9	0.78	− 4.3	− 1.0	− 2.3	− 5.1

*POST* postbiotic, *PRO* probiotic, *PLA* placebo

*Denotes significance

**Table 4:** Time of maximal observed concentration (*T*_max_)

Amino acid	Postbiotic		Probiotic		Placebo		*p* (Friedman test)	Median percent differences	
	Median	IQR	Median	IQR	Median	IQR		POST vs. PRO	PRO vs. PLA
Alanine	120	60	60	67.5	45	120	0.14	90.9	28.6
Arginine	120	60	120	60	120	60	0.89	0.0	0.0
Asparagine	120	60	60	60	60	60	0.37	66.7	0.0
Aspartic Acid	150	150	45	90	60	67.5	0.41	85.7	− 28.6
BCAA	120	30	120	60	120	15	0.58	0.0	0.0
Citrulline	45	97.5	60	52.5	30	60	0.21	40.0	66.7
Cystine	0	75	0	60	0	30	0.38	NA	NA
EAA	120	75	120	60	120	60	0.70	0.0	0.0
Glutamic Acid	60	105	60	90	45	97.5	0.50	28.6	28.6
Glutamine	90	67.5	60	97.5	60	97.5	0.49	40.0	0.0
Glycine	120	75	60	90	60	90	0.19	66.7	0.0
Histidine	120	60	60	60	60	90	0.23	66.7	0.0
Isoleucine	120	60	120	15	120	60	0.03*	0.0	0.0
Leucine	120	75	120	15	120	60	0.96	0.0	0.0
Lysine	120	75	120	60	120	60	0.13	0.0	0.0
Methionine	60	97.5	60	75	30	60	0.04*	66.7	66.7
Ornithine	120	60	120	60	120	15	0.03*	0.0	0.0
Phenylalanine	120	60	90	60	90	60	0.08	28.6	0.0
Proline	90	60	60	60	60	45	0.06	40.0	0.0
Serine	120	120	60	67.5	90	67.5	0.41	28.6	− 40.0
Threonine	120	75	90	60	60	60	0.09	66.7	40.0
Total AA	120	30	90	60	120	60	0.04*	0.0	− 28.6
Tryptophan	60	97.5	60	37.5	30	75	0.25	66.7	66.7
Tyrosine	120	60	120	60	90	60	0.63	28.6	28.6
Valine	120	15	90	60	120	15	0.48	0.0	− 28.6

*POST* postbiotic, *PRO* probiotic, *PLA* placebo

*Denotes significance

### Safety Analysis

All safety blood values for CBC and CMP were within normal limits for all participants throughout the study duration. Safety outcomes are displayed in the supplementary material.

## Discussion

The increasing worldwide interest in adopting a more plant-based, whole-food diet is often met with concerns about whether these diets allow for the ingestion of adequate protein, both in terms of quantity and quality. A lifestyle implementing a balanced plant-based and protein-rich diet can offer health benefits for individuals [30]; however, the distribution of amino acids within plant protein versus animal protein differs and may subsequently impact the gut—even when protein consumption meets the recommended dietary allowance (RDA) [31]. Probiotics as a whole have demonstrated the ability to aid human physiology in multiple ways including the improvement of gut health [11], mood [32], stress and depression [33], immune system function [34], and allergy response [35]. Various strains of probiotics have been observed to improve the absorption of nutrients, namely, protein, through their influence in upregulating digestive enzymes, which has led some researchers to believe that probiotics may also positively influence body composition by facilitating muscle protein synthesis [20, 36, 37].

Postbiotics are a more recent conception and are thought to positively influence health as effectively as probiotics with differences only in their preparation, though research is warranted to fully elucidate the mechanisms by which postbiotics affect the gut [8]. Additionally, the potential benefits of postbiotic supplementation could extend beyond just the host. For example, postbiotics are not as sensitive as their “live” counterparts and are therefore less susceptible to degradation with changes in the environment. This characteristic may allow for a longer shelf-life and facilitate transport and storage without deterioration. Postbiotics may also possess the ability to better withstand the harsh environment of the digestive tract [8].

In a previous study, we demonstrated that the ingestion of plant protein with 2 weeks of probiotic supplementation containing *L. paracasei* DG and *L. paracasei* LPC-S01 increased circulating levels of amino acids when compared to a placebo. Thus, the purpose of the present study was to examine whether supplementation of a multi-strain probiotic consisting of 5 billion CFU *L. paracasei* DG plus 5 billion CFU *L. paracasei* LPC-S01 or a postbiotic consisting of the same strains would alter the absorption of individual and total amino acids following ingestion of a plant-based meal. The primary findings of the present study were observed in the postbiotic supplementation group whereby the observed increase in circulating levels of alanine, asparagine, citrulline, cystine, glycine, methionine, proline, and total amino acids demonstrates: (1) confirmation that the administration of the strains DG and LPC-S01 may aid in protein absorption and (2) that postbiotic supplementation *L. paracasei* DG and LPC-S01 may potentially be more effective than probiotic supplementation. Moreover, incremental area under the curve (iAUC) values were higher for arginine in the postbiotic condition when compared to the placebo condition. Additionally, time to concentration max (*T*_max_) in the postbiotic condition occurred later than in both the placebo and probiotic conditions for isoleucine, methionine, ornithine, and total amino acids. This may reflect additional unique effects of postbiotics on intestinal physiology.

Prior to the present investigation, there were, to our knowledge, no studies that examined postbiotic supplementation and its co-ingestion of a plant protein–based meal. That said, the current body of knowledge regarding postbiotic supplementation and its positive impact on health cannot be ignored. Postbiotics have been reported in animal models to modulate the gut environment leading to subsequent benefits similar to those obtained through probiotic supplementation [38–40]. Investigations analyzing postbiotics in humans have reached similar conclusions relative to the efficacy of postbiotics. Lee and colleagues [41] assessed probiotic versus postbiotic supplementation compared to a placebo following 6 weeks of treatment and measured biomarkers indicative of muscle damage. They observed that the postbiotic was just as effective in reducing muscle damage as probiotics when compared to the placebo. In fact, there are several studies that observe the positive influence of postbiotics whether it is immune function, recovery, inflammation, and now, plant protein absorption [41–44].

In our study, we selected a postbiotic prepared through γ-irradiation over the more commonly used heat inactivation. Recent studies indicate that gamma irradiation removes lactobacilli’s ability to replicate while preserving their metabolic activity [45]. Similarly, in our study, tests on the industrial biomass of *L. paracasei* LPC-S01 demonstrated that γ-irradiation not only effectively inactivates cells’ reproductive capacity but also preserves both membrane integrity, potentially leading to better retention of the probiotic properties of the original live microorganism. The preservation of membrane integrity is essential for bacterial cells because it maintains the proper internal environment for cellular functions, including enzyme activity. If the membrane is compromised, it can disrupt enzyme function.

Unexpectedly, we observed that irradiated bacterial cells were even more effective than live cells in facilitating the release of free amino acids. This surprising and apparently counterintuitive result could have several speculative explanations. One possibility is related to *L. paracasei*’s auxotrophy for various amino acids, which, as in many lactobacilli, is due to evolutionary adaptation to nutrient-rich substrates like milk or animal intestines, leading to efficient systems for the import and use of exogenous amino acids [46–48]. Unlike live bacteria, irradiated cells are unable to reproduce, likely resulting in reduced amino acid import and utilization, which in turn may leave a greater pool of free amino acids available for host absorption. It is also possible that γ-inactivation, despite preserving cytoplasmic membranes, induces the release of cellular components such as proteolytic enzymes, which could further promote protein hydrolysis. Finally, we cannot rule out that the postbiotic and the probiotic preparations may influence differently the host’s microbiota, immune responses, or gene expression in the small intestine mucosa, thereby impacting amino acid absorption. These hypotheses, however, remain speculative and require further investigation. Overall, the present study adds to the mounting evidence in support of postbiotic supplementation and offers the first observation of improved amino acid absorption following the co-ingestion of a postbiotic supplement versus a probiotic supplement with a plant protein–based, mixed meal.

Interestingly, in this trial, the probiotic condition exhibited increases in only cystine (+ 9%) when compared to the placebo. This finding differs from our previous experiment in which the appearance of amino acids following the co-ingestion of a probiotic supplement and plant protein was greater in multiple amino acids, including total amino acids, when compared to placebo [20]. Specifically, increases in leucine (+ 23.3%), isoleucine (+ 26.0%), valine (+ 21.5%), and total EAA (+ 16.0%) were observed in the probiotic condition as compared to the placebo condition [20]. A possible explanation for these differences could be due to the selected protein source and the amino acid composition of those sources. Despite protein sources from both studies containing similar amounts of pea protein (20 g), the vegan patty selected in the present investigation included an additional 14 g of fat and 7 g of carbohydrate. The mixed-meal aspect of this investigation may have altered absorption times in comparison to the protein shake absorption kinetics which was seemingly unaffected in the absence of carbohydrates and fat [20]. Yoshii and colleagues [49] observed that co-ingestion of leucine with a mixed meal containing fat, protein, and carbohydrates suppressed the appearance of leucine in plasma. Unfortunately, this was the only amino acid the investigators measured so comparisons to other individual amino acids, other BCAAs, EAAs, and total amino acids cannot be extrapolated.

Future research into postbiotic influence on nutrient absorption should continue and efforts to identify their mechanisms of action are necessary. Although probiotics can influence absorption through several different mechanisms, it is purported that increased digestive enzyme production is one of the primary drivers of amino acid absorption. The postbiotic used in this investigation was produced with membrane integrity intact and maintained enzyme activity; therefore, we speculate that amino acid absorption improved through increased digestive enzyme activity elicited by the postbiotic. This investigation presents promising and novel evidence that postbiotic supplementation enhances amino acid absorption in a plant-based, mixed meal. Additional research into varying forms of protein and amino acid profiles should also be conducted to fully understand the influence of postbiotics. For example, examination of the appearance of amino acids following the co-ingestion of a postbiotic with a whole food source of protein (plant versus animal), protein powder (plant versus whey versus soy), and mixed meals (animal versus plant-based) would aid in deepening our understanding. Finally, of interest to athletes and other individuals who choose to adopt a plant-based diet is the absorption of BCAAs and EAAs to optimize muscle repair and muscle protein synthesis. Evidence already exists demonstrating the ability of postbiotics to influence recovery; therefore, additional research into specific populations (e.g., vegans or vegetarians) should be conducted to determine if postbiotic supplements can similarly influence muscle growth and recovery.

A limitation of this research was the inability to analyze the intestinal microbiome to determine whether the interventions affected the abundance of microbial taxa or their metabolites. Although a follow-up study in women is expected, we recognize that the inclusion of only men is a limitation.

## Conclusions

Two weeks of supplementation of postbiotic supplementation containing 5 billion AFU *L. paracasei* DG plus 5 billion AFU *L. paracasei* LPC-S01 resulted in significant improvements in amino acid absorption profiles for various amino acids and total amino acids compared to placebo and probiotic supplementation. This is the first data to report an improved absorption of amino acids in a mixed macronutrient meal and provides a rationale for probiotic and/or postbiotic supplementation as a support strategy to improve the amino acid response in the post-prandial state.

## Supplementary Information

Below is the link to the electronic supplementary material.

Supplementary file1 (PDF 457 kb)

Supplementary file2 (XLSX 14 kb)

Supplementary file3 (DOCX 50 kb)

Supplementary file4 (DOCX 50 kb)

Supplementary file5 (XLSX 17 kb)

## Data Availability

No datasets were generated or analysed during the current study.

## References

[1] Hill C, Guarner F, Reid G, Gibson GR, Merenstein DJ, Pot B et al (2014) The international scientific association for probiotics and prebiotics consensus statement on the scope and appropriate use of the term probiotic. Nat Rev Gastroenterol Hepatol 11(8):506–514. 10.1038/nrgastro.2014.66 24912386 10.1038/nrgastro.2014.66

[2] Jäger R, Mohr AE, Carpenter KC, Kerksick CM, Purpura M, Moussa A et al (2019) International society of sports nutrition position stand: probiotics. J Int Soc Sports Nutr 16(1):62. 10.1186/s12970-019-0329-0 31864419 10.1186/s12970-019-0329-0 PMC6925426

[3] Jäger R, Shields KA, Lowery RP, De Souza EO, Partl JM, Hollmer C et al (2016) Probiotic Bacillus coagulans GBI-30, 6086 reduces exercise-induced muscle damage and increases recovery. PeerJ 4:e2276. 10.7717/peerj.2276 27547577 10.7717/peerj.2276 PMC4963221

[4] Fiore W, Arioli S, Guglielmetti S (2020) The Neglected microbial components of commercial probiotic formulations. Microorganisms 8(8):1177 32756409 10.3390/microorganisms8081177 PMC7464440

[5] Salminen S, Collado MC, Endo A, Hill C, Lebeer S, Quigley EMM et al (2021) The International Scientific Association of Probiotics and Prebiotics (ISAPP) consensus statement on the definition and scope of postbiotics. Nat Rev Gastroenterol Hepatol 18(9):649–667. 10.1038/s41575-021-00440-6 33948025 10.1038/s41575-021-00440-6 PMC8387231

[6] Asif A, Afzaal M, Shahid H, Saeed F, Ahmed A, Shah YA et al (2023) Probing the functional and therapeutic properties of postbiotics in relation to their industrial application. Food Sci Nutr 11(8):4472–4484. 10.1002/fsn3.3465 37576043 10.1002/fsn3.3465 PMC10420781

[7] Aguilar-Toalá JE, Arioli S, Behare P, Belzer C, BerniCanani R, Chatel J-M et al (2021) Postbiotics — when simplification fails to clarify. Nat Rev Gastroenterol Hepatol 18(11):825–826. 10.1038/s41575-021-00521-6 34556825 10.1038/s41575-021-00521-6

[8] Vinderola G, Sanders ME, Salminen S (2022) The concept of postbiotics. Foods 11(8). 10.3390/foods11081077 10.3390/foods11081077 PMC9027423 35454664

[9] Guglielmetti S (2023) Safety considerations in the use of nonviable microbial cells as health-promoting agents in food and dietary supplements. Curr Opin Food Sci 54:101105. 10.1016/j.cofs.2023.101105

[10] Tarrerias AL, Costil V, Vicari F, Létard JC, Adenis-Lamarre P, Aisène A et al (2011) The effect of inactivated Lactobacillus LB fermented culture medium on symptom severity: observational investigation in 297 patients with diarrhea-predominant irritable bowel syndrome. Dig Dis 29(6):588–591. 10.1159/000332987 22179215 10.1159/000332987

[11] Clarke SF, Murphy EF, O’Sullivan O, Lucey AJ, Humphreys M, Hogan A et al (2014) Exercise and associated dietary extremes impact on gut microbial diversity. Gut 63(12):1913–1920. 10.1136/gutjnl-2013-306541 25021423 10.1136/gutjnl-2013-306541

[12] Gleeson M, Bishop NC, Oliveira M, Tauler P (2011) Daily probiotic’s (Lactobacillus casei Shirota) reduction of infection incidence in athletes. Int J Sport Nutr Exerc Metab 21(1):55–64. 10.1123/ijsnem.21.1.55 21411836 10.1123/ijsnem.21.1.55

[13] Haywood BA, Black KE, Baker D, McGarvey J, Healey P, Brown RC (2014) Probiotic supplementation reduces the duration and incidence of infections but not severity in elite rugby union players. J Sci Med Sport 17(4):356–360. 10.1016/j.jsams.2013.08.004 24045086 10.1016/j.jsams.2013.08.004

[14] Maathuis AJ, Keller D, Farmer S (2010) Survival and metabolic activity of the GanedenBC30 strain of Bacillus coagulans in a dynamic in vitro model of the stomach and small intestine. Benef Microbes 1(1):31–36. 10.3920/bm2009.0009 21831748 10.3920/BM2009.0009

[15] Jahan-Mihan A, Luhovyy BL, El Khoury D, Anderson GH (2011) Dietary proteins as determinants of metabolic and physiologic functions of the gastrointestinal tract. Nutrients 3(5):574–603. 10.3390/nu3050574 22254112 10.3390/nu3050574 PMC3257691

[16] Ferrari L, Panaite SA, Bertazzo A, Visioli F (2022) Animal- and plant-based protein sources: a scoping review of human health outcomes and environmental impact. Nutrients 14(23). 10.3390/nu14235115 10.3390/nu14235115 PMC9741334 36501146

[17] Garaus M, Garaus C (2023) US consumers’ mental associations with meat substitute products. Front Nutr 10:1135476. 10.3389/fnut.2023.1135476 37051122 10.3389/fnut.2023.1135476 PMC10083498

[18] Alcorta A, Porta A, Tárrega A, Alvarez MD, Vaquero MP (2021) Foods for plant-based diets: challenges and innovations. Foods 10(2). 10.3390/foods10020293 10.3390/foods10020293 PMC7912826 33535684

[19] Aschemann-Witzel J, Gantriis RF, Fraga P, Perez-Cueto FJA (2021) Plant-based food and protein trend from a business perspective: markets, consumers, and the challenges and opportunities in the future. Crit Rev Food Sci Nutr 61(18):3119–3128. 10.1080/10408398.2020.1793730 32654499 10.1080/10408398.2020.1793730

[20] Jäger R, Zaragoza J, Purpura M, Iametti S, Marengo M, Tinsley GM et al (2020) Probiotic administration increases amino acid absorption from plant protein: a placebo-controlled, randomized, double-blind, multicenter, crossover study. Probiotics Antimicrob Proteins 12(4):1330–1339. 10.1007/s12602-020-09656-5 32358640 10.1007/s12602-020-09656-5 PMC7641926

[21] Brunelli L, De Vitis V, Ferrari R, Minuzzo M, Fiore W, Jäger R et al (2022) In vitro assessment of the probiotic properties of an industrial preparation containing Lacticaseibacillus paracasei in the context of athlete health. Front Pharmacol 13:857987. 10.3389/fphar.2022.857987 36016576 10.3389/fphar.2022.857987 PMC9397523

[22] Ferrario C, Taverniti V, Milani C, Fiore W, Laureati M, De Noni I et al (2014) Modulation of fecal Clostridiales bacteria and butyrate by probiotic intervention with Lactobacillus paracasei DG varies among healthy adults. J Nutr 144(11):1787–1796. 10.3945/jn.114.197723 25332478 10.3945/jn.114.197723

[23] Radicioni M, Koirala R, Fiore W, Leuratti C, Guglielmetti S, Arioli S (2019) Survival of L. casei DG(®) (Lactobacillus paracasei CNCMI1572) in the gastrointestinal tract of a healthy paediatric population. Eur J Nutr 58(8):3161–70. 10.1007/s00394-018-1860-5 30498868 10.1007/s00394-018-1860-5 PMC6842349

[24] Arioli S, Koirala R, Taverniti V, Fiore W, Guglielmetti S (2018) Quantitative recovery of viable Lactobacillus paracasei CNCM I-1572 (L. casei DG®) after gastrointestinal passage in healthy adults. Front Microbiol. 9:1720. 10.3389/fmicb.2018.01720 30116228 10.3389/fmicb.2018.01720 PMC6083036

[25] Pinheiro J, Bates D, DebRoy S, Sarkar D, R. Core Team (2021) Software: nlme - linear and nonlinear mixed effects models

[26] Lüdecke D (2021) Software: sjPlot - data visualization for statistics in social science

[27] Brouns F, Bjorck I, Frayn KN, Gibbs AL, Lang V, Slama G et al (2005) Glycaemic index methodology. Nutr Res Rev 18(1):145–171. 10.1079/nrr2005100 19079901 10.1079/NRR2005100

[28] Denney WS, Duvvuri S, Buckeridge C (2015) Simple, Automatic noncompartmental analysis: the PKNCA R package. J Pharmacokinet Pharmacodynam 42(1):11–107,S65. 10.1007/s10928-015-9432-2

[29] Kassambara A (2020) rstatix: pipe-friendly framework for basic statistical tests

[30] Mariotti F, Gardner CD (2019) Dietary protein and amino acids in vegetarian diets-a review. Nutrients 11(11). 10.3390/nu11112661 10.3390/nu11112661 PMC6893534 31690027

[31] Wang F, Wan Y, Yin K, Wei Y, Wang B, Yu X et al (2019) Lower circulating branched-chain amino acid concentrations among vegetarians are associated with changes in gut microbial composition and function. Mol Nutr Food Res 63(24):e1900612. 10.1002/mnfr.201900612 31703241 10.1002/mnfr.201900612

[32] Marotta A, Sarno E, Del Casale A, Pane M, Mogna L, Amoruso A et al (2019) Effects of probiotics on cognitive reactivity, mood, and sleep quality. Front Psychiatry 10:164. 10.3389/fpsyt.2019.00164 30971965 10.3389/fpsyt.2019.00164 PMC6445894

[33] Walden KE, Moon JM, Hagele AM, Allen LE, Gaige CJ, Krieger JM et al (2023) A randomized controlled trial to examine the impact of a multi-strain probiotic on self-reported indicators of depression, anxiety, mood, and associated biomarkers. Front Nutr 10:1219313. 10.3389/fnut.2023.1219313 37720373 10.3389/fnut.2023.1219313 PMC10501394

[34] Mazziotta C, Tognon M, Martini F, Torreggiani E, Rotondo JC (2023) Probiotics mechanism of action on immune cells and beneficial effects on human health. Cells 12(1). 10.3390/cells12010184 10.3390/cells12010184 PMC9818925 36611977

[35] Huang J, Zhang J, Wang X, Jin Z, Zhang P, Su H et al (2022) Effect of probiotics on respiratory tract allergic disease and gut microbiota. Front Nutr 9:821900. 10.3389/fnut.2022.821900 35295917 10.3389/fnut.2022.821900 PMC8920559

[36] Jäger R, Purpura M, Farmer S, Cash HA, Keller D (2018) Probiotic Bacillus coagulans GBI-30, 6086 improves protein absorption and utilization. Probiotics Antimicrob Proteins 10(4):611–615. 10.1007/s12602-017-9354-y 29196920 10.1007/s12602-017-9354-y PMC6208742

[37] Toohey JC, Townsend JR, Johnson SB, Toy AM, Vantrease WC, Bender D et al (2020) Effects of probiotic (Bacillus subtilis) supplementation during offseason resistance training in female division I athletes. J Strength Condition Res 34(11):3173–3181. 10.1519/jsc.0000000000002675 10.1519/JSC.0000000000002675 33105368

[38] Warda AK, Rea K, Fitzgerald P, Hueston C, Gonzalez-Tortuero E, Dinan TG et al (2019) Heat-killed lactobacilli alter both microbiota composition and behaviour. Behav Brain Res 362:213–223. 10.1016/j.bbr.2018.12.047 30597248 10.1016/j.bbr.2018.12.047

[39] Hsieh FC, Lan CC, Huang TY, Chen KW, Chai CY, Chen WT et al (2016) Heat-killed and live Lactobacillus reuteri GMNL-263 exhibit similar effects on improving metabolic functions in high-fat diet-induced obese rats. Food Funct 7(5):2374–2388. 10.1039/c5fo01396h 27163114 10.1039/c5fo01396h

[40] Wu Y, Wang Y, Hu A, Shu X, Huang W, Liu J et al (2022) Lactobacillus plantarum-derived postbiotics prevent Salmonella-induced neurological dysfunctions by modulating gut-brain axis in mice. Front Nutr 9:946096. 10.3389/fnut.2022.946096 35967771 10.3389/fnut.2022.946096 PMC9365972

[41] Lee MC, Ho CS, Hsu YJ, Huang CC (2022) Live and heat-killed probiotic Lactobacillus paracasei PS23 accelerated the improvement and recovery of strength and damage biomarkers after exercise-induced muscle damage. Nutrients 14(21). 10.3390/nu14214563 10.3390/nu14214563 PMC9658587 36364825

[42] Lee CC, Liao YC, Lee MC, Cheng YC, Chiou SY, Lin JS et al. (2022) Different impacts of heat-killed and viable Lactiplantibacillus plantarum TWK10 on exercise performance, fatigue, body composition, and gut microbiota in humans. Microorganisms 10(11). 10.3390/microorganisms10112181 10.3390/microorganisms10112181 PMC9692508 36363775

[43] Asama T, Kimura Y, Kono T, Tatefuji T, Hashimoto K, Benno Y (2016) Effects of heat-killed Lactobacillus kunkeei YB38 on human intestinal environment and bowel movement: a pilot study. Benef Microbes 7(3):337–344. 10.3920/bm2015.0132 26839076 10.3920/BM2015.0132

[44] Kato K, Arai S, Sato S, Iwabuchi N, Takara T, Tanaka M (2024) Effects of heat-killed Lacticaseibacillus paracasei MCC1849 on immune parameters in healthy adults-a randomized, double-blind, placebo-controlled, parallel-group study. Nutrients 16(2). 10.3390/nu16020216 10.3390/nu16020216 PMC10821487 38257109

[45] Porfiri L, Burtscher J, Kangethe RT, Verhovsek D, Cattoli G, Domig KJ et al. (2022) Irradiated non-replicative lactic acid bacteria preserve metabolic activity while exhibiting diverse immune modulation. Front Veter Sci 9. 10.3389/fvets.2022.859124 10.3389/fvets.2022.859124 PMC9158532 35664846

[46] Ardö Y (2006) Flavour formation by amino acid catabolism. Biotechnol Adv 24(2):238–242. 10.1016/j.biotechadv.2005.11.005 16406465 10.1016/j.biotechadv.2005.11.005

[47] Bringel F, Hubert J-C (2003) Extent of genetic lesions of the arginine and pyrimidine biosynthetic pathways in Lactobacillus plantarum, L. paraplantarum, L. pentosus, and L. casei: prevalence of CO2-dependent auxotrophs and characterization of deficient arg genes in L. plantarum. Appl Environ Microbiol 69(5):2674–83. 10.1128/AEM.69.5.2674-2683.2003 12732536 10.1128/AEM.69.5.2674-2683.2003 PMC154521

[48] Kiousi DE, Efstathiou C, Tegopoulos K, Mantzourani I, Alexopoulos A, Plessas S et al. (2022) Genomic insight into Lacticaseibacillus paracasei SP5, reveals genes and gene clusters of probiotic interest and biotechnological potential. Front Microbiol 13. 10.3389/fmicb.2022.922689 10.3389/fmicb.2022.922689 PMC9244547 35783439

[49] Yoshii N, Sato K, Ogasawara R, Nishimura Y, Shinohara Y, Fujita S (2018) Effect of mixed meal and leucine intake on plasma amino acid concentrations in young men. Nutrients 10(10). 10.3390/nu10101543 10.3390/nu10101543 PMC6213454 30340425

