# Supplementary material for: Postbiotic Supplementation Increases Amino Acid Absorption from Plant-Based Meal: A Placebo-Controlled, Randomized, Double-Blind, Crossover Study
Source: Probiotics Antimicrob Proteins. 2025 Feb 24;17(5):3641–55. doi: 10.1007/s12602-025-10480-y (PMC12532647; doi:10.1007/s12602-025-10480-y)

## SUPPLEMENTARY INFORMATION

## FIGURE 1. PERCENT CHANGES IN AMINO ACIDS

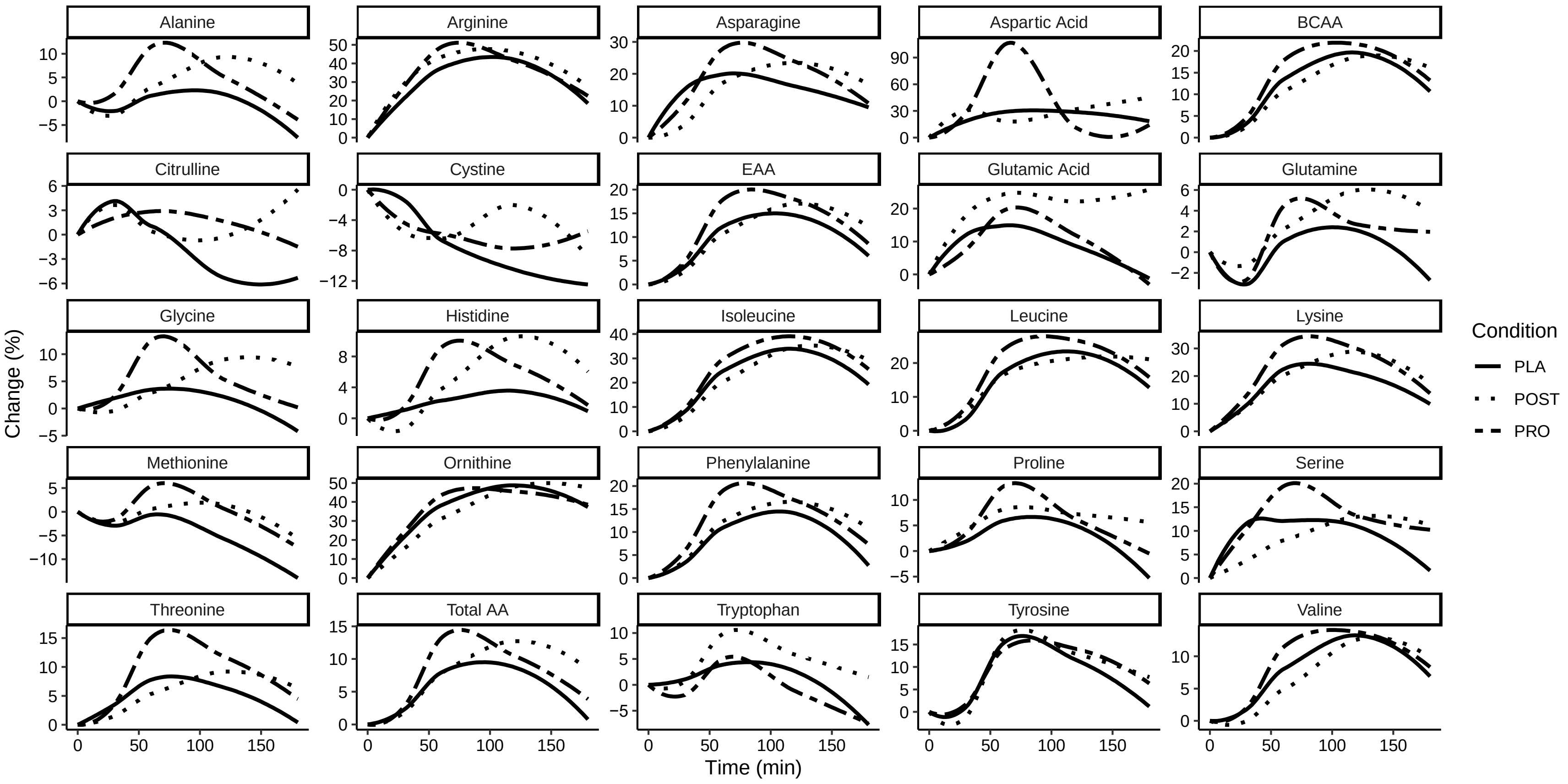

## FIGURE 2. CHANGES IN RAW AMINO ACID CONCENTRATIONS

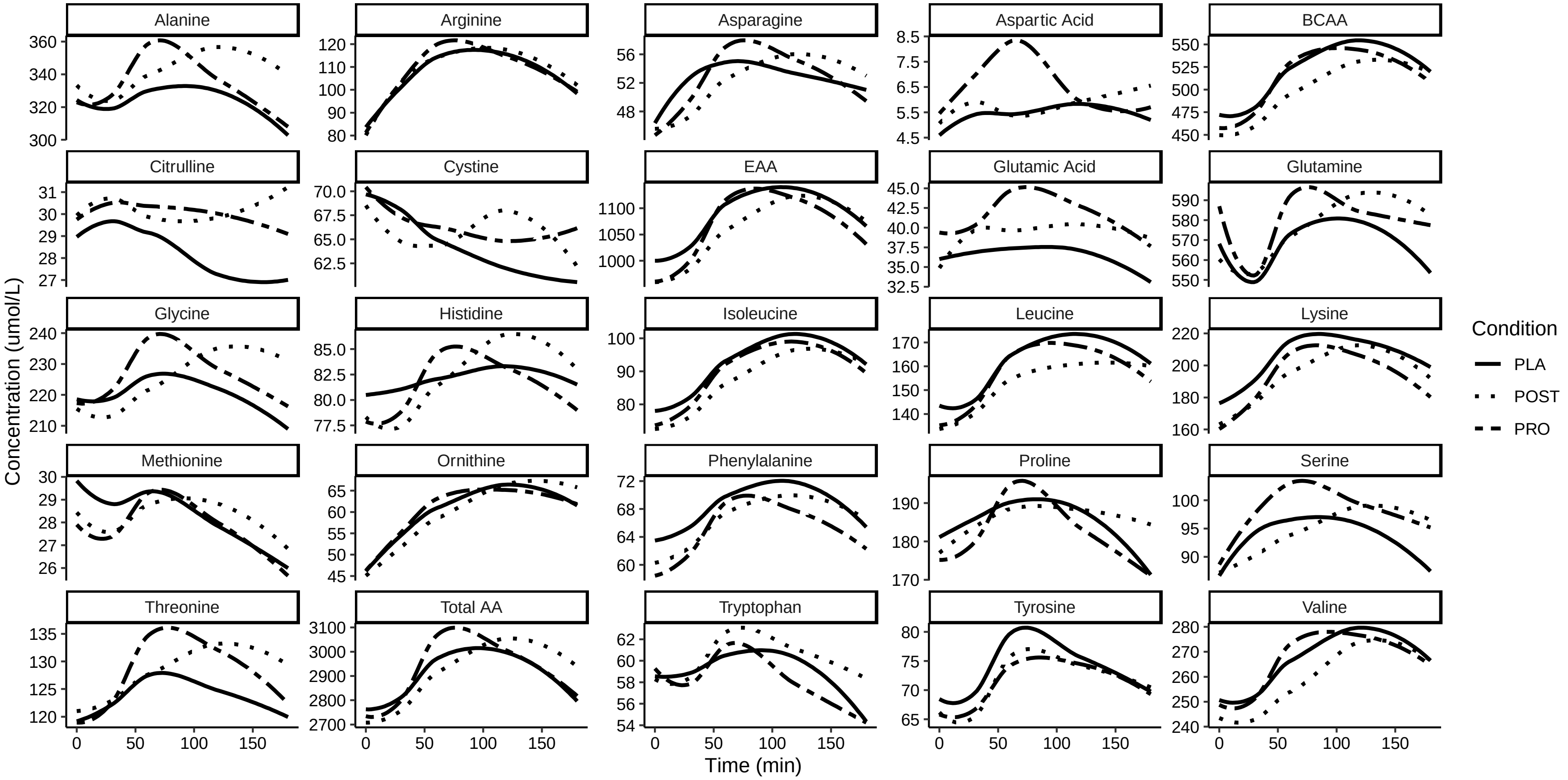

# FIGURE 3. INCREMENTAL AREA UNDER THE CURVE (IAUC) BOXPLOTS

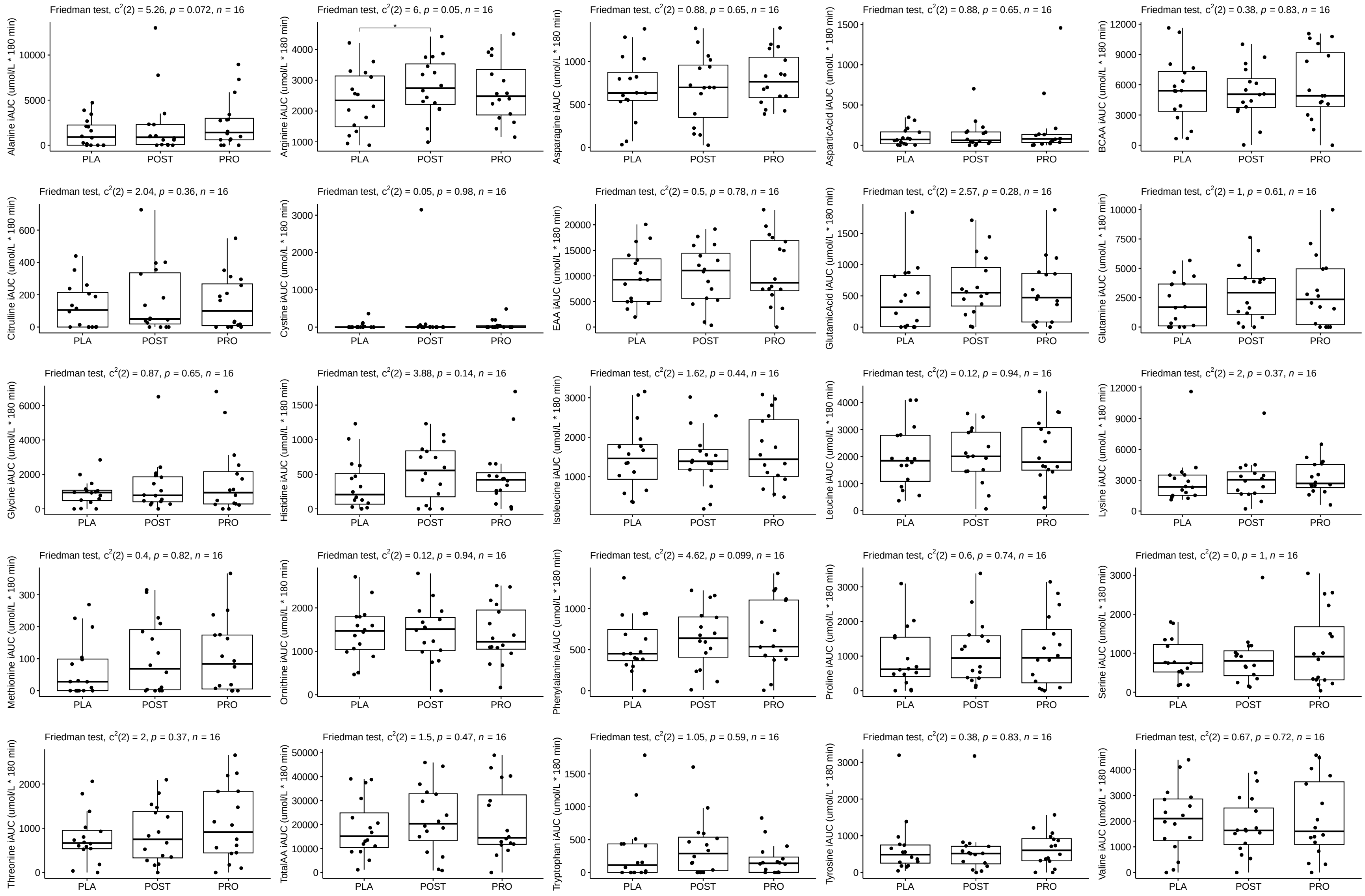

# FIGURE 4. MAXIMAL OBSERVED CONCENTRATION (CMAX) BOXPLOTS

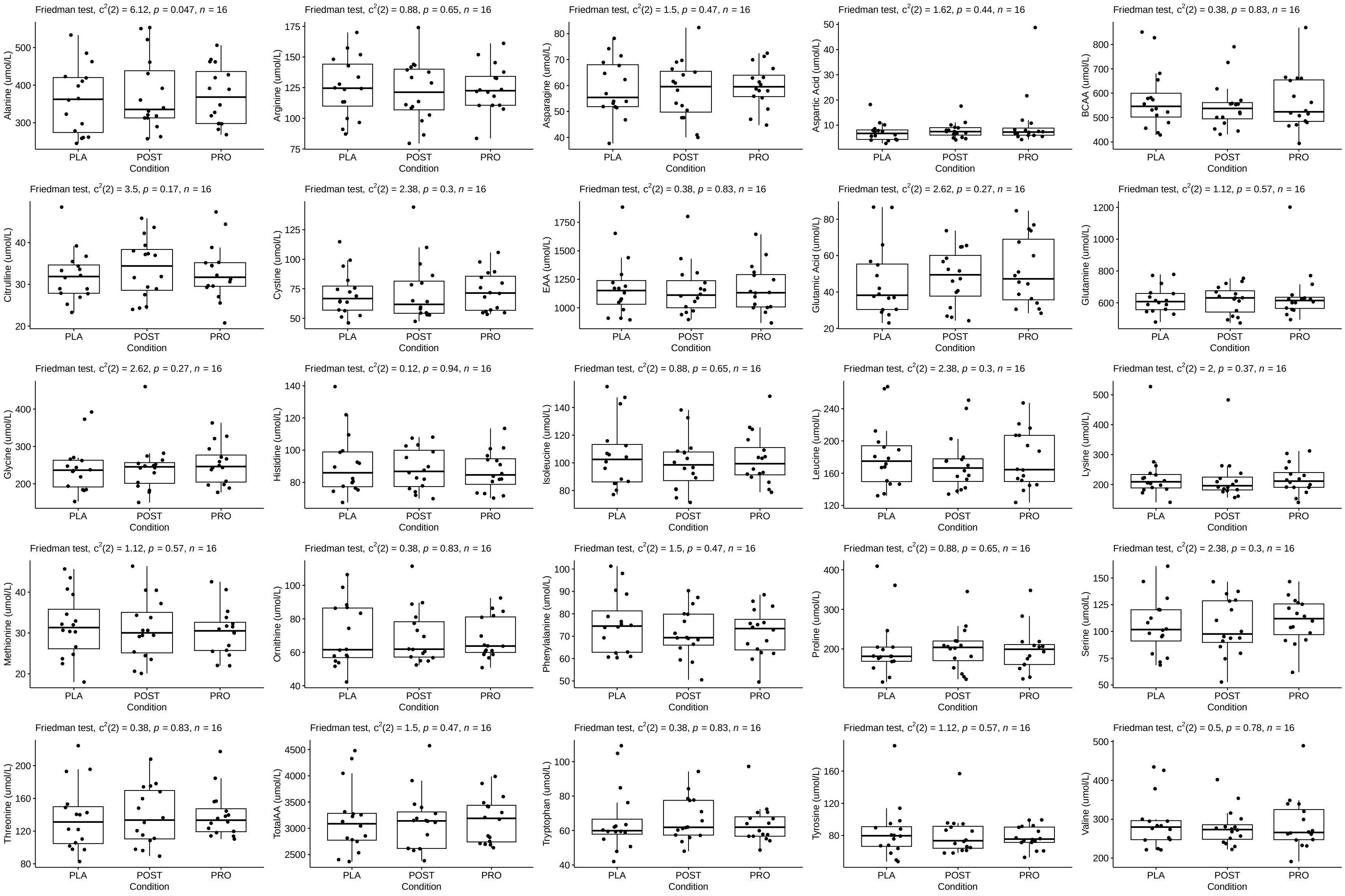

# FIGURE 5. TIME OF MAXIMAL OBSERVED CONCENTRATION (TMAX) BOXPLOTS

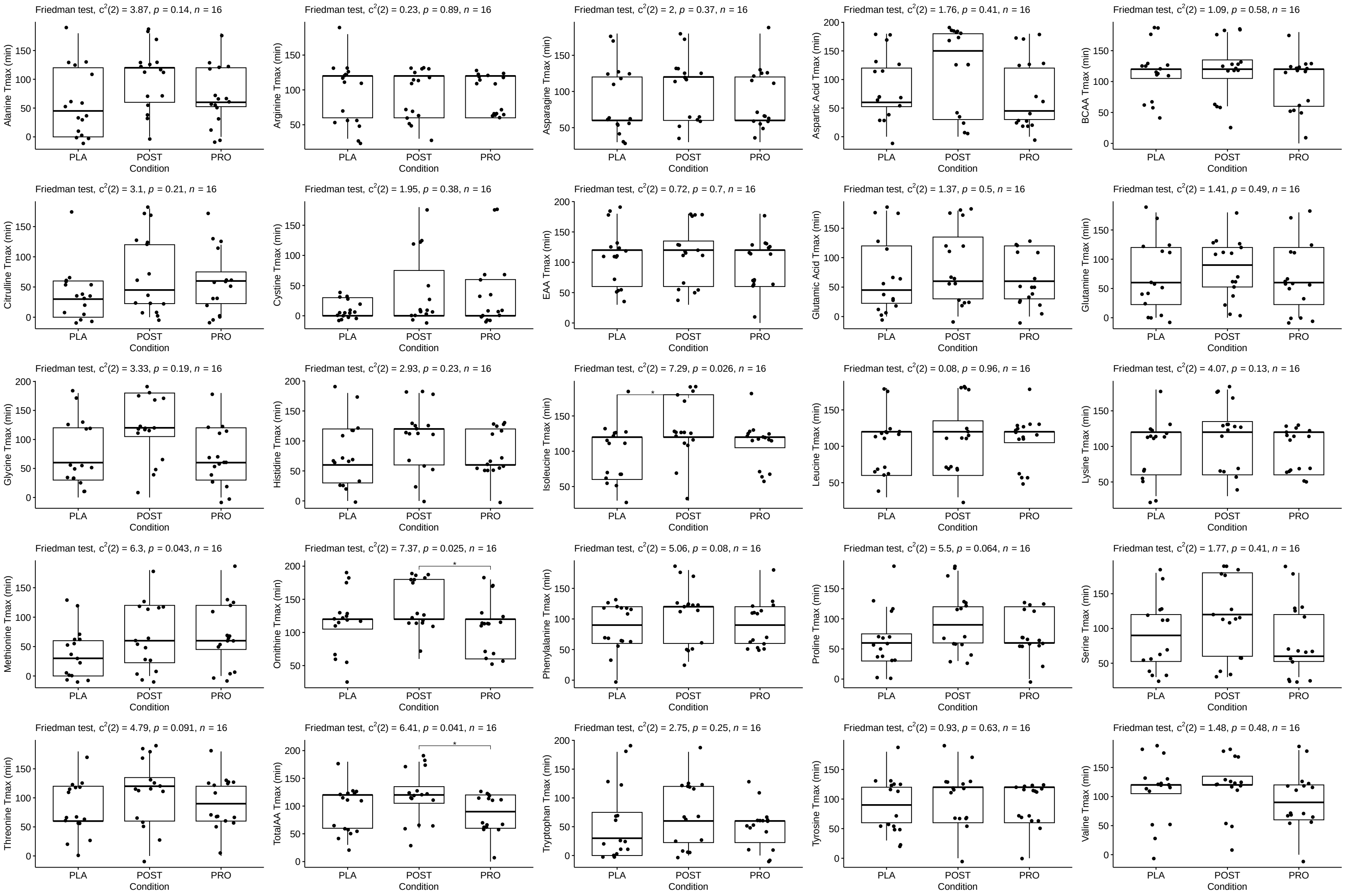

Supplement: Supplementary file 1 — Supplementary file1 (PDF 457 kb) [file 12602_2025_10480_MOESM1_ESM.pdf]
