# Supplementary material for: Postbiotic Supplementation Increases Amino Acid Absorption from Plant-Based Meal: A Placebo-Controlled, Randomized, Double-Blind, Crossover Study
Source: Probiotics Antimicrob Proteins. 2025 Feb 24;17(5):3641–55. doi: 10.1007/s12602-025-10480-y (PMC12532647; doi:10.1007/s12602-025-10480-y)
Supplement: Supplementary file 3 — Supplementary file3 (DOCX 50 kb) [file 12602_2025_10480_MOESM3_ESM.docx]

|  | **Alanine** | | |
| --- | --- | --- | --- |
| *Predictors* | *Estimates* | *CI* | *p* |
| (Intercept) | 0.98 | -4.07 – 6.03 | 0.703 |
| Condition [POST] | -1.60 | -7.83 – 4.64 | 0.614 |
| Condition [PRO] | 3.61 | -2.62 – 9.85 | 0.255 |
| interval | -0.03 | -0.07 – 0.01 | 0.165 |
| Condition [POST] × interval | 0.07 | 0.01 – 0.13 | **0.022** |
| Condition [PRO] × interval | 0.01 | -0.05 – 0.07 | 0.785 |
| **Random Effects** | | | |
| σ^2^ | 163.05 | | |
| τ_00_ _ID_ | 24.91 | | |
| N _ID_ | 16 | | |
| Observations | 240 | | |
| Marginal R^2^ / Conditional R^2^ | 0.048 / NA | | |

|  | **Arginine** | | |
| --- | --- | --- | --- |
| *Predictors* | *Estimates* | *CI* | *p* |
| (Intercept) | 16.55 | 8.49 – 24.60 | **<0.001** |
| Condition [POST] | 3.10 | -8.30 – 14.49 | 0.593 |
| Condition [PRO] | 4.71 | -6.68 – 16.10 | 0.416 |
| interval | 0.09 | 0.01 – 0.17 | **0.021** |
| Condition [POST] × interval | 0.03 | -0.08 – 0.14 | 0.604 |
| Condition [PRO] × interval | -0.01 | -0.12 – 0.11 | 0.916 |
| **Random Effects** | | | |
| σ^2^ | 543.94 | | |
| τ_00_ _ID_ | 0.00 | | |
| ICC | 0.00 | | |
| N _ID_ | 16 | | |
| Observations | 240 | | |
| Marginal R^2^ / Conditional R^2^ | 0.083 / 0.083 | | |

|  | **Asparagine** | | |
| --- | --- | --- | --- |
| *Predictors* | *Estimates* | *CI* | *p* |
| (Intercept) | 10.08 | 4.79 – 15.36 | **<0.001** |
| Condition [POST] | -5.95 | -12.89 – 1.00 | 0.093 |
| Condition [PRO] | 0.61 | -6.33 – 7.56 | 0.862 |
| interval | 0.03 | -0.02 – 0.07 | 0.285 |
| Condition [POST] × interval | 0.08 | 0.01 – 0.15 | **0.026** |
| Condition [PRO] × interval | 0.02 | -0.04 – 0.09 | 0.481 |
| **Random Effects** | | | |
| σ^2^ | 202.14 | | |
| τ_00_ _ID_ | 15.72 | | |
| N _ID_ | 16 | | |
| Observations | 240 | | |
| Marginal R^2^ / Conditional R^2^ | 0.095 / NA | | |

|  | **AsparticAcid** | | |
| --- | --- | --- | --- |
| *Predictors* | *Estimates* | *CI* | *p* |
| (Intercept) | 12.63 | -26.48 – 51.74 | 0.525 |
| Condition [POST] | -2.75 | -57.94 – 52.43 | 0.922 |
| Condition [PRO] | 23.20 | -31.98 – 78.38 | 0.408 |
| interval | 0.08 | -0.30 – 0.47 | 0.681 |
| Condition [POST] × interval | 0.12 | -0.43 – 0.66 | 0.672 |
| Condition [PRO] × interval | -0.14 | -0.68 – 0.40 | 0.614 |
| **Random Effects** | | | |
| σ^2^ | 12764.50 | | |
| τ_00_ _ID_ | 27.74 | | |
| N _ID_ | 16 | | |
| Observations | 240 | | |
| Marginal R^2^ / Conditional R^2^ | 0.007 / NA | | |

|  | **BCAA** | | |
| --- | --- | --- | --- |
| *Predictors* | *Estimates* | *CI* | *p* |
| (Intercept) | 3.74 | -1.21 – 8.70 | 0.138 |
| Condition [POST] | -2.07 | -7.51 – 3.38 | 0.455 |
| Condition [PRO] | 1.30 | -4.14 – 6.75 | 0.638 |
| interval | 0.07 | 0.03 – 0.11 | **<0.001** |
| Condition [POST] × interval | 0.03 | -0.02 – 0.08 | 0.292 |
| Condition [PRO] × interval | 0.01 | -0.04 – 0.06 | 0.728 |
| **Random Effects** | | | |
| σ^2^ | 124.34 | | |
| τ_00_ _ID_ | 39.99 | | |
| N _ID_ | 16 | | |
| Observations | 240 | | |
| Marginal R^2^ / Conditional R^2^ | 0.204 / NA | | |

|  | **Citrulline** | | |
| --- | --- | --- | --- |
| *Predictors* | *Estimates* | *CI* | *p* |
| (Intercept) | 2.59 | -2.92 – 8.09 | 0.356 |
| Condition [POST] | -2.10 | -7.44 – 3.24 | 0.440 |
| Condition [PRO] | -0.71 | -6.05 – 4.63 | 0.794 |
| interval | -0.05 | -0.08 – -0.01 | **0.014** |
| Condition [POST] × interval | 0.06 | 0.01 – 0.12 | **0.016** |
| Condition [PRO] × interval | 0.04 | -0.02 – 0.09 | 0.181 |
| **Random Effects** | | | |
| σ^2^ | 119.64 | | |
| τ_00_ _ID_ | 66.11 | | |
| N _ID_ | 16 | | |
| Observations | 240 | | |
| Marginal R^2^ / Conditional R^2^ | 0.042 / NA | | |

|  | **Cystine** | | |
| --- | --- | --- | --- |
| *Predictors* | *Estimates* | *CI* | *p* |
| (Intercept) | -0.50 | -3.35 – 2.36 | 0.732 |
| Condition [POST] | -1.80 | -5.57 – 1.98 | 0.349 |
| Condition [PRO] | -2.05 | -5.83 – 1.72 | 0.285 |
| interval | -0.07 | -0.10 – -0.05 | **<0.001** |
| Condition [POST] × interval | 0.04 | 0.01 – 0.08 | **0.021** |
| Condition [PRO] × interval | 0.05 | 0.01 – 0.08 | **0.016** |
| **Random Effects** | | | |
| σ^2^ | 59.73 | | |
| τ_00_ _ID_ | 4.26 | | |
| N _ID_ | 16 | | |
| Observations | 240 | | |
| Marginal R^2^ / Conditional R^2^ | 0.146 / NA | | |

|  | **EAA** | | |
| --- | --- | --- | --- |
| *Predictors* | *Estimates* | *CI* | *p* |
| (Intercept) | 4.21 | 0.13 – 8.29 | **0.043** |
| Condition [POST] | -1.74 | -6.77 – 3.29 | 0.496 |
| Condition [PRO] | 1.63 | -3.40 – 6.66 | 0.524 |
| interval | 0.04 | 0.00 – 0.07 | **0.028** |
| Condition [POST] × interval | 0.04 | -0.01 – 0.09 | 0.132 |
| Condition [PRO] × interval | 0.01 | -0.04 – 0.06 | 0.649 |
| **Random Effects** | | | |
| σ^2^ | 106.12 | | |
| τ_00_ _ID_ | 16.42 | | |
| N _ID_ | 16 | | |
| Observations | 240 | | |
| Marginal R^2^ / Conditional R^2^ | 0.126 / NA | | |

|  | **GlutamicAcid** | | |
| --- | --- | --- | --- |
| *Predictors* | *Estimates* | *CI* | *p* |
| (Intercept) | 9.10 | -3.52 – 21.72 | 0.157 |
| Condition [POST] | 0.45 | -14.73 – 15.62 | 0.954 |
| Condition [PRO] | -0.06 | -15.23 – 15.12 | 0.994 |
| interval | -0.03 | -0.13 – 0.08 | 0.592 |
| Condition [POST] × interval | 0.14 | -0.01 – 0.29 | 0.072 |
| Condition [PRO] × interval | 0.00 | -0.15 – 0.15 | 0.957 |
| **Random Effects** | | | |
| σ^2^ | 965.35 | | |
| τ_00_ _ID_ | 181.91 | | |
| N _ID_ | 16 | | |
| Observations | 240 | | |
| Marginal R^2^ / Conditional R^2^ | 0.045 / NA | | |

|  | **Glutamine** | | |
| --- | --- | --- | --- |
| *Predictors* | *Estimates* | *CI* | *p* |
| (Intercept) | -0.32 | -4.29 – 3.64 | 0.872 |
| Condition [POST] | -0.01 | -4.96 – 4.95 | 0.998 |
| Condition [PRO] | 0.25 | -4.70 – 5.20 | 0.921 |
| interval | -0.00 | -0.04 – 0.03 | 0.872 |
| Condition [POST] × interval | 0.04 | -0.01 – 0.08 | 0.158 |
| Condition [PRO] × interval | 0.02 | -0.03 – 0.07 | 0.418 |
| **Random Effects** | | | |
| σ^2^ | 102.83 | | |
| τ_00_ _ID_ | 14.11 | | |
| N _ID_ | 16 | | |
| Observations | 240 | | |
| Marginal R^2^ / Conditional R^2^ | 0.030 / NA | | |

|  | **Glycine** | | |
| --- | --- | --- | --- |
| *Predictors* | *Estimates* | *CI* | *p* |
| (Intercept) | 2.49 | -2.30 – 7.28 | 0.306 |
| Condition [POST] | -2.89 | -8.69 – 2.92 | 0.328 |
| Condition [PRO] | 1.88 | -3.93 – 7.69 | 0.525 |
| interval | -0.02 | -0.06 – 0.02 | 0.253 |
| Condition [POST] × interval | 0.08 | 0.02 – 0.14 | **0.008** |
| Condition [PRO] × interval | 0.02 | -0.04 – 0.08 | 0.506 |
| **Random Effects** | | | |
| σ^2^ | 141.39 | | |
| τ_00_ _ID_ | 25.02 | | |
| N _ID_ | 16 | | |
| Observations | 240 | | |
| Marginal R^2^ / Conditional R^2^ | 0.049 / NA | | |

|  | **Histidine** | | |
| --- | --- | --- | --- |
| *Predictors* | *Estimates* | *CI* | *p* |
| (Intercept) | 1.00 | -2.73 – 4.73 | 0.597 |
| Condition [POST] | -1.16 | -6.17 – 3.85 | 0.648 |
| Condition [PRO] | 1.97 | -3.04 – 6.98 | 0.439 |
| interval | 0.01 | -0.03 – 0.04 | 0.685 |
| Condition [POST] × interval | 0.04 | -0.01 – 0.09 | 0.084 |
| Condition [PRO] × interval | 0.00 | -0.05 – 0.05 | 0.878 |
| **Random Effects** | | | |
| σ^2^ | 105.15 | | |
| τ_00_ _ID_ | 5.55 | | |
| N _ID_ | 16 | | |
| Observations | 240 | | |
| Marginal R^2^ / Conditional R^2^ | 0.045 / NA | | |

|  | **Isoleucine** | | |
| --- | --- | --- | --- |
| *Predictors* | *Estimates* | *CI* | *p* |
| (Intercept) | 7.64 | -0.49 – 15.77 | 0.065 |
| Condition [POST] | -3.79 | -12.30 – 4.72 | 0.381 |
| Condition [PRO] | 0.82 | -7.68 – 9.33 | 0.849 |
| interval | 0.12 | 0.06 – 0.18 | **<0.001** |
| Condition [POST] × interval | 0.06 | -0.02 – 0.14 | 0.165 |
| Condition [PRO] × interval | 0.03 | -0.05 – 0.12 | 0.424 |
| **Random Effects** | | | |
| σ^2^ | 303.45 | | |
| τ_00_ _ID_ | 123.24 | | |
| N _ID_ | 16 | | |
| Observations | 240 | | |
| Marginal R^2^ / Conditional R^2^ | 0.253 / NA | | |

|  | **Leucine** | | |
| --- | --- | --- | --- |
| *Predictors* | *Estimates* | *CI* | *p* |
| (Intercept) | 4.58 | -1.32 – 10.49 | 0.128 |
| Condition [POST] | -0.97 | -7.69 – 5.74 | 0.775 |
| Condition [PRO] | 2.65 | -4.07 – 9.37 | 0.438 |
| interval | 0.09 | 0.04 – 0.13 | **<0.001** |
| Condition [POST] × interval | 0.03 | -0.03 – 0.10 | 0.332 |
| Condition [PRO] × interval | 0.01 | -0.06 – 0.07 | 0.807 |
| **Random Effects** | | | |
| σ^2^ | 189.17 | | |
| τ_00_ _ID_ | 50.65 | | |
| N _ID_ | 16 | | |
| Observations | 240 | | |
| Marginal R^2^ / Conditional R^2^ | 0.191 / NA | | |

|  | **Lysine** | | |
| --- | --- | --- | --- |
| *Predictors* | *Estimates* | *CI* | *p* |
| (Intercept) | 8.75 | 3.04 – 14.45 | **0.003** |
| Condition [POST] | -1.82 | -9.31 – 5.67 | 0.632 |
| Condition [PRO] | 3.39 | -4.10 – 10.87 | 0.374 |
| interval | 0.05 | -0.00 – 0.10 | 0.064 |
| Condition [POST] × interval | 0.05 | -0.02 – 0.13 | 0.149 |
| Condition [PRO] × interval | 0.02 | -0.05 – 0.09 | 0.580 |
| **Random Effects** | | | |
| σ^2^ | 234.90 | | |
| τ_00_ _ID_ | 18.48 | | |
| N _ID_ | 16 | | |
| Observations | 240 | | |
| Marginal R^2^ / Conditional R^2^ | 0.112 / NA | | |

|  | **Methionine** | | |
| --- | --- | --- | --- |
| *Predictors* | *Estimates* | *CI* | *p* |
| (Intercept) | 0.99 | -2.40 – 4.39 | 0.564 |
| Condition [POST] | -0.62 | -5.19 – 3.96 | 0.790 |
| Condition [PRO] | 1.22 | -3.36 – 5.80 | 0.600 |
| interval | -0.07 | -0.10 – -0.04 | **<0.001** |
| Condition [POST] × interval | 0.05 | 0.01 – 0.10 | **0.023** |
| Condition [PRO] × interval | 0.04 | -0.01 – 0.08 | 0.118 |
| **Random Effects** | | | |
| σ^2^ | 87.78 | | |
| τ_00_ _ID_ | 4.29 | | |
| N _ID_ | 16 | | |
| Observations | 240 | | |
| Marginal R^2^ / Conditional R^2^ | 0.129 / NA | | |

|  | **Ornithine** | | |
| --- | --- | --- | --- |
| *Predictors* | *Estimates* | *CI* | *p* |
| (Intercept) | 13.76 | 4.27 – 23.25 | **0.005** |
| Condition [POST] | -6.05 | -16.93 – 4.82 | 0.274 |
| Condition [PRO] | 2.09 | -8.79 – 12.96 | 0.706 |
| interval | 0.20 | 0.12 – 0.27 | **<0.001** |
| Condition [POST] × interval | 0.07 | -0.04 – 0.18 | 0.214 |
| Condition [PRO] × interval | -0.01 | -0.12 – 0.10 | 0.837 |
| **Random Effects** | | | |
| σ^2^ | 495.67 | | |
| τ_00_ _ID_ | 127.56 | | |
| N _ID_ | 16 | | |
| Observations | 240 | | |
| Marginal R^2^ / Conditional R^2^ | 0.290 / NA | | |

|  | **Phenylalanine** | | |
| --- | --- | --- | --- |
| *Predictors* | *Estimates* | *CI* | *p* |
| (Intercept) | 4.29 | -0.06 – 8.63 | 0.053 |
| Condition [POST] | -0.82 | -6.02 – 4.38 | 0.756 |
| Condition [PRO] | 2.43 | -2.77 – 7.63 | 0.359 |
| interval | 0.02 | -0.01 – 0.06 | 0.186 |
| Condition [POST] × interval | 0.04 | -0.01 – 0.09 | 0.105 |
| Condition [PRO] × interval | 0.02 | -0.04 – 0.07 | 0.565 |
| **Random Effects** | | | |
| σ^2^ | 113.45 | | |
| τ_00_ _ID_ | 21.86 | | |
| N _ID_ | 16 | | |
| Observations | 240 | | |
| Marginal R^2^ / Conditional R^2^ | 0.092 / NA | | |

|  | **Proline** | | |
| --- | --- | --- | --- |
| *Predictors* | *Estimates* | *CI* | *p* |
| (Intercept) | 3.45 | -0.41 – 7.31 | 0.079 |
| Condition [POST] | -0.45 | -5.26 – 4.37 | 0.854 |
| Condition [PRO] | 1.52 | -3.29 – 6.34 | 0.534 |
| interval | -0.03 | -0.06 – 0.01 | 0.140 |
| Condition [POST] × interval | 0.05 | 0.00 – 0.10 | **0.035** |
| Condition [PRO] × interval | 0.02 | -0.03 – 0.06 | 0.487 |
| **Random Effects** | | | |
| σ^2^ | 97.21 | | |
| τ_00_ _ID_ | 13.48 | | |
| N _ID_ | 16 | | |
| Observations | 240 | | |
| Marginal R^2^ / Conditional R^2^ | 0.042 / NA | | |

|  | **Serine** | | |
| --- | --- | --- | --- |
| *Predictors* | *Estimates* | *CI* | *p* |
| (Intercept) | 7.82 | 1.85 – 13.78 | **0.010** |
| Condition [POST] | -5.75 | -13.69 – 2.19 | 0.155 |
| Condition [PRO] | 0.00 | -7.94 – 7.95 | 0.999 |
| interval | -0.01 | -0.06 – 0.05 | 0.798 |
| Condition [POST] × interval | 0.07 | -0.01 – 0.15 | 0.069 |
| Condition [PRO] × interval | 0.04 | -0.03 – 0.12 | 0.276 |
| **Random Effects** | | | |
| σ^2^ | 264.34 | | |
| τ_00_ _ID_ | 16.57 | | |
| N _ID_ | 16 | | |
| Observations | 240 | | |
| Marginal R^2^ / Conditional R^2^ | 0.038 / NA | | |

|  | **Threonine** | | |
| --- | --- | --- | --- |
| *Predictors* | *Estimates* | *CI* | *p* |
| (Intercept) | 3.61 | -0.20 – 7.41 | 0.063 |
| Condition [POST] | -2.35 | -7.30 – 2.59 | 0.350 |
| Condition [PRO] | 1.43 | -3.52 – 6.38 | 0.569 |
| interval | 0.00 | -0.03 – 0.03 | 0.994 |
| Condition [POST] × interval | 0.04 | -0.01 – 0.09 | 0.098 |
| Condition [PRO] × interval | 0.02 | -0.02 – 0.07 | 0.327 |
| **Random Effects** | | | |
| σ^2^ | 102.60 | | |
| τ_00_ _ID_ | 9.14 | | |
| N _ID_ | 16 | | |
| Observations | 240 | | |
| Marginal R^2^ / Conditional R^2^ | 0.048 / NA | | |

|  | **TotalAA** | | |
| --- | --- | --- | --- |
| *Predictors* | *Estimates* | *CI* | *p* |
| (Intercept) | 3.23 | -0.09 – 6.54 | 0.056 |
| Condition [POST] | -1.41 | -5.82 – 3.00 | 0.529 |
| Condition [PRO] | 1.10 | -3.32 – 5.51 | 0.625 |
| interval | 0.01 | -0.02 – 0.04 | 0.557 |
| Condition [POST] × interval | 0.05 | 0.00 – 0.09 | **0.036** |
| Condition [PRO] × interval | 0.01 | -0.03 – 0.06 | 0.555 |
| **Random Effects** | | | |
| σ^2^ | 81.59 | | |
| τ_00_ _ID_ | 5.17 | | |
| N _ID_ | 16 | | |
| Observations | 240 | | |
| Marginal R^2^ / Conditional R^2^ | 0.071 / NA | | |

|  | **Tryptophan** | | |
| --- | --- | --- | --- |
| *Predictors* | *Estimates* | *CI* | *p* |
| (Intercept) | 2.95 | -1.70 – 7.60 | 0.212 |
| Condition [POST] | -0.09 | -6.18 – 5.99 | 0.976 |
| Condition [PRO] | -1.07 | -7.15 – 5.02 | 0.730 |
| interval | -0.04 | -0.08 – 0.00 | 0.077 |
| Condition [POST] × interval | 0.05 | -0.01 – 0.11 | 0.123 |
| Condition [PRO] × interval | -0.00 | -0.06 – 0.06 | 0.991 |
| **Random Effects** | | | |
| σ^2^ | 155.14 | | |
| τ_00_ _ID_ | 12.74 | | |
| N _ID_ | 16 | | |
| Observations | 240 | | |
| Marginal R^2^ / Conditional R^2^ | 0.051 / NA | | |

|  | **Tyrosine** | | |
| --- | --- | --- | --- |
| *Predictors* | *Estimates* | *CI* | *p* |
| (Intercept) | 4.73 | -1.11 – 10.56 | 0.112 |
| Condition [POST] | -1.69 | -9.66 – 6.28 | 0.676 |
| Condition [PRO] | -0.96 | -8.93 – 7.01 | 0.813 |
| interval | 0.01 | -0.04 – 0.07 | 0.631 |
| Condition [POST] × interval | 0.04 | -0.04 – 0.12 | 0.331 |
| Condition [PRO] × interval | 0.03 | -0.05 – 0.11 | 0.455 |
| **Random Effects** | | | |
| σ^2^ | 266.00 | | |
| τ_00_ _ID_ | 9.65 | | |
| N _ID_ | 16 | | |
| Observations | 240 | | |
| Marginal R^2^ / Conditional R^2^ | 0.026 / NA | | |

|  | **Valine** | | |
| --- | --- | --- | --- |
| *Predictors* | *Estimates* | *CI* | *p* |
| (Intercept) | 2.14 | -1.77 – 6.04 | 0.282 |
| Condition [POST] | -2.09 | -6.47 – 2.30 | 0.349 |
| Condition [PRO] | 0.77 | -3.61 – 5.16 | 0.728 |
| interval | 0.05 | 0.02 – 0.08 | **0.002** |
| Condition [POST] × interval | 0.02 | -0.02 – 0.06 | 0.368 |
| Condition [PRO] × interval | 0.00 | -0.04 – 0.05 | 0.832 |
| **Random Effects** | | | |
| σ^2^ | 80.50 | | |
| τ_00_ _ID_ | 23.20 | | |
| N _ID_ | 16 | | |
| Observations | 240 | | |
| Marginal R^2^ / Conditional R^2^ | 0.154 / NA | | |
