# Supplementary material for: Postbiotic Supplementation Increases Amino Acid Absorption from Plant-Based Meal: A Placebo-Controlled, Randomized, Double-Blind, Crossover Study
Source: Probiotics Antimicrob Proteins. 2025 Feb 24;17(5):3641–55. doi: 10.1007/s12602-025-10480-y (PMC12532647; doi:10.1007/s12602-025-10480-y)
Supplement: Supplementary file 4 — Supplementary file4 (DOCX 50 kb) [file 12602_2025_10480_MOESM4_ESM.docx]

|  | **Alanine** | | |
| --- | --- | --- | --- |
| *Predictors* | *Estimates* | *CI* | *p* |
| (Intercept) | 327.92 | 293.61 – 362.23 | **<0.001** |
| Condition [POST] | 3.27 | -25.23 – 31.77 | 0.821 |
| Condition [PRO] | 9.42 | -19.08 – 37.92 | 0.516 |
| time | -0.09 | -0.29 – 0.11 | 0.391 |
| Condition [POST] × time | 0.18 | -0.10 – 0.46 | 0.208 |
| Condition [PRO] × time | 0.00 | -0.28 – 0.28 | 0.991 |
| **Random Effects** | | | |
| σ^2^ | 3404.50 | | |
| τ_00_ _ID_ | 3175.44 | | |
| N _ID_ | 16 | | |
| Observations | 240 | | |
| Marginal R^2^ / Conditional R^2^ | 0.024 / NA | | |

|  | **Arginine** | | |
| --- | --- | --- | --- |
| *Predictors* | *Estimates* | *CI* | *p* |
| (Intercept) | 97.14 | 87.37 – 106.91 | **<0.001** |
| Condition [POST] | -1.59 | -10.95 – 7.76 | 0.738 |
| Condition [PRO] | 0.94 | -8.41 – 10.30 | 0.843 |
| time | 0.07 | 0.00 – 0.13 | **0.037** |
| Condition [POST] × time | 0.03 | -0.06 – 0.12 | 0.516 |
| Condition [PRO] × time | 0.00 | -0.09 – 0.09 | 0.979 |
| **Random Effects** | | | |
| σ^2^ | 366.81 | | |
| τ_00_ _ID_ | 212.88 | | |
| N _ID_ | 16 | | |
| Observations | 240 | | |
| Marginal R^2^ / Conditional R^2^ | 0.071 / NA | | |

|  | **Asparagine** | | |
| --- | --- | --- | --- |
| *Predictors* | *Estimates* | *CI* | *p* |
| (Intercept) | 50.53 | 46.42 – 54.65 | **<0.001** |
| Condition [POST] | -3.34 | -6.97 – 0.30 | 0.072 |
| Condition [PRO] | -1.09 | -4.73 – 2.55 | 0.556 |
| time | 0.01 | -0.01 – 0.04 | 0.260 |
| Condition [POST] × time | 0.03 | -0.00 – 0.07 | 0.074 |
| Condition [PRO] × time | 0.01 | -0.03 – 0.04 | 0.663 |
| **Random Effects** | | | |
| σ^2^ | 55.48 | | |
| τ_00_ _ID_ | 42.60 | | |
| N _ID_ | 16 | | |
| Observations | 240 | | |
| Marginal R^2^ / Conditional R^2^ | 0.071 / NA | | |

|  | **AsparticAcid** | | |
| --- | --- | --- | --- |
| *Predictors* | *Estimates* | *CI* | *p* |
| (Intercept) | 5.07 | 3.58 – 6.56 | **<0.001** |
| Condition [POST] | 0.16 | -1.53 – 1.86 | 0.849 |
| Condition [PRO] | 1.68 | -0.01 – 3.38 | 0.052 |
| time | 0.00 | -0.01 – 0.01 | 0.637 |
| Condition [POST] × time | 0.00 | -0.01 – 0.02 | 0.637 |
| Condition [PRO] × time | -0.01 | -0.02 – 0.01 | 0.443 |
| **Random Effects** | | | |
| σ^2^ | 12.05 | | |
| τ_00_ _ID_ | 3.21 | | |
| N _ID_ | 16 | | |
| Observations | 240 | | |
| Marginal R^2^ / Conditional R^2^ | 0.027 / NA | | |

|  | **BCAA** | | |
| --- | --- | --- | --- |
| *Predictors* | *Estimates* | *CI* | *p* |
| (Intercept) | 483.75 | 432.24 – 535.26 | **<0.001** |
| Condition [POST] | -27.44 | -57.73 – 2.85 | 0.076 |
| Condition [PRO] | -6.22 | -36.51 – 24.07 | 0.686 |
| time | 0.34 | 0.12 – 0.55 | **0.002** |
| Condition [POST] × time | 0.11 | -0.19 – 0.40 | 0.487 |
| Condition [PRO] × time | -0.02 | -0.32 – 0.28 | 0.916 |
| **Random Effects** | | | |
| σ^2^ | 3844.90 | | |
| τ_00_ _ID_ | 9039.58 | | |
| N _ID_ | 16 | | |
| Observations | 240 | | |
| Marginal R^2^ / Conditional R^2^ | 0.142 / NA | | |

|  | **Citrulline** | | |
| --- | --- | --- | --- |
| *Predictors* | *Estimates* | *CI* | *p* |
| (Intercept) | 29.57 | 26.57 – 32.56 | **<0.001** |
| Condition [POST] | 0.42 | -1.46 – 2.29 | 0.662 |
| Condition [PRO] | 0.77 | -1.11 – 2.64 | 0.421 |
| time | -0.01 | -0.03 – -0.00 | **0.027** |
| Condition [POST] × time | 0.02 | 0.00 – 0.04 | **0.043** |
| Condition [PRO] × time | 0.01 | -0.01 – 0.03 | 0.295 |
| **Random Effects** | | | |
| σ^2^ | 14.71 | | |
| τ_00_ _ID_ | 29.72 | | |
| N _ID_ | 16 | | |
| Observations | 240 | | |
| Marginal R^2^ / Conditional R^2^ | 0.067 / NA | | |

|  | **Cystine** | | |
| --- | --- | --- | --- |
| *Predictors* | *Estimates* | *CI* | *p* |
| (Intercept) | 69.13 | 60.44 – 77.82 | **<0.001** |
| Condition [POST] | -2.04 | -5.23 – 1.16 | 0.211 |
| Condition [PRO] | -0.46 | -3.66 – 2.73 | 0.775 |
| time | -0.05 | -0.07 – -0.03 | **<0.001** |
| Condition [POST] × time | 0.03 | 0.00 – 0.06 | **0.043** |
| Condition [PRO] × time | 0.03 | -0.00 – 0.06 | 0.054 |
| **Random Effects** | | | |
| σ^2^ | 42.83 | | |
| τ_00_ _ID_ | 290.08 | | |
| N _ID_ | 16 | | |
| Observations | 240 | | |
| Marginal R^2^ / Conditional R^2^ | 0.118 / NA | | |

|  | **EAA** | | |
| --- | --- | --- | --- |
| *Predictors* | *Estimates* | *CI* | *p* |
| (Intercept) | 1033.03 | 930.52 – 1135.54 | **<0.001** |
| Condition [POST] | -51.69 | -114.87 – 11.50 | 0.108 |
| Condition [PRO] | -21.89 | -85.08 – 41.29 | 0.495 |
| time | 0.44 | 0.00 – 0.89 | **0.048** |
| Condition [POST] × time | 0.31 | -0.32 – 0.93 | 0.333 |
| Condition [PRO] × time | -0.01 | -0.63 – 0.62 | 0.987 |
| **Random Effects** | | | |
| σ^2^ | 16733.34 | | |
| τ_00_ _ID_ | 35063.85 | | |
| N _ID_ | 16 | | |
| Observations | 240 | | |
| Marginal R^2^ / Conditional R^2^ | 0.081 / NA | | |

|  | **GlutamicAcid** | | |
| --- | --- | --- | --- |
| *Predictors* | *Estimates* | *CI* | *p* |
| (Intercept) | 37.27 | 30.12 – 44.42 | **<0.001** |
| Condition [POST] | 0.24 | -4.24 – 4.72 | 0.916 |
| Condition [PRO] | 4.32 | -0.16 – 8.80 | 0.059 |
| time | -0.02 | -0.05 – 0.02 | 0.339 |
| Condition [POST] × time | 0.03 | -0.01 – 0.07 | 0.185 |
| Condition [PRO] × time | 0.01 | -0.04 – 0.05 | 0.763 |
| **Random Effects** | | | |
| σ^2^ | 84.19 | | |
| τ_00_ _ID_ | 169.11 | | |
| N _ID_ | 16 | | |
| Observations | 240 | | |
| Marginal R^2^ / Conditional R^2^ | 0.052 / NA | | |

|  | **Glutamine** | | |
| --- | --- | --- | --- |
| *Predictors* | *Estimates* | *CI* | *p* |
| (Intercept) | 565.22 | 526.85 – 603.60 | **<0.001** |
| Condition [POST] | -7.04 | -41.52 – 27.44 | 0.688 |
| Condition [PRO] | 11.47 | -23.01 – 45.95 | 0.513 |
| time | -0.01 | -0.25 – 0.23 | 0.944 |
| Condition [POST] × time | 0.19 | -0.15 – 0.53 | 0.275 |
| Condition [PRO] × time | 0.03 | -0.31 – 0.37 | 0.851 |
| **Random Effects** | | | |
| σ^2^ | 4983.15 | | |
| τ_00_ _ID_ | 3617.22 | | |
| N _ID_ | 16 | | |
| Observations | 240 | | |
| Marginal R^2^ / Conditional R^2^ | 0.016 / NA | | |

|  | **Glycine** | | |
| --- | --- | --- | --- |
| *Predictors* | *Estimates* | *CI* | *p* |
| (Intercept) | 222.60 | 196.53 – 248.68 | **<0.001** |
| Condition [POST] | -8.37 | -21.92 – 5.18 | 0.225 |
| Condition [PRO] | 2.59 | -10.96 – 16.14 | 0.707 |
| time | -0.05 | -0.14 – 0.05 | 0.327 |
| Condition [POST] × time | 0.16 | 0.03 – 0.30 | **0.018** |
| Condition [PRO] × time | 0.04 | -0.10 – 0.17 | 0.589 |
| **Random Effects** | | | |
| σ^2^ | 769.88 | | |
| τ_00_ _ID_ | 2423.13 | | |
| N _ID_ | 16 | | |
| Observations | 240 | | |
| Marginal R^2^ / Conditional R^2^ | 0.034 / NA | | |

|  | **Histidine** | | |
| --- | --- | --- | --- |
| *Predictors* | *Estimates* | *CI* | *p* |
| (Intercept) | 81.06 | 75.42 – 86.69 | **<0.001** |
| Condition [POST] | -2.78 | -7.69 – 2.14 | 0.266 |
| Condition [PRO] | -1.02 | -5.93 – 3.90 | 0.684 |
| time | 0.01 | -0.03 – 0.04 | 0.646 |
| Condition [POST] × time | 0.03 | -0.02 – 0.08 | 0.214 |
| Condition [PRO] × time | 0.00 | -0.05 – 0.05 | 0.994 |
| **Random Effects** | | | |
| σ^2^ | 101.20 | | |
| τ_00_ _ID_ | 81.08 | | |
| N _ID_ | 16 | | |
| Observations | 240 | | |
| Marginal R^2^ / Conditional R^2^ | 0.024 / NA | | |

|  | **Isoleucine** | | |
| --- | --- | --- | --- |
| *Predictors* | *Estimates* | *CI* | *p* |
| (Intercept) | 82.07 | 72.76 – 91.39 | **<0.001** |
| Condition [POST] | -6.99 | -13.61 – -0.37 | **0.039** |
| Condition [PRO] | -3.05 | -9.67 – 3.57 | 0.365 |
| time | 0.09 | 0.05 – 0.14 | **<0.001** |
| Condition [POST] × time | 0.03 | -0.03 – 0.10 | 0.356 |
| Condition [PRO] × time | 0.01 | -0.06 – 0.07 | 0.864 |
| **Random Effects** | | | |
| σ^2^ | 183.77 | | |
| τ_00_ _ID_ | 267.03 | | |
| N _ID_ | 16 | | |
| Observations | 240 | | |
| Marginal R^2^ / Conditional R^2^ | 0.216 / NA | | |

|  | **Leucine** | | |
| --- | --- | --- | --- |
| *Predictors* | *Estimates* | *CI* | *p* |
| (Intercept) | 148.00 | 131.27 – 164.74 | **<0.001** |
| Condition [POST] | -9.90 | -20.08 – 0.28 | 0.057 |
| Condition [PRO] | -3.88 | -14.06 – 6.30 | 0.453 |
| time | 0.12 | 0.05 – 0.19 | **0.001** |
| Condition [POST] × time | 0.03 | -0.07 – 0.13 | 0.588 |
| Condition [PRO] × time | -0.01 | -0.11 – 0.09 | 0.842 |
| **Random Effects** | | | |
| σ^2^ | 434.37 | | |
| τ_00_ _ID_ | 939.77 | | |
| N _ID_ | 16 | | |
| Observations | 240 | | |
| Marginal R^2^ / Conditional R^2^ | 0.158 / NA | | |

|  | **Lysine** | | |
| --- | --- | --- | --- |
| *Predictors* | *Estimates* | *CI* | *p* |
| (Intercept) | 189.74 | 161.69 – 217.78 | **<0.001** |
| Condition [POST] | -15.93 | -33.60 – 1.74 | 0.077 |
| Condition [PRO] | -11.37 | -29.04 – 6.30 | 0.206 |
| time | 0.12 | -0.00 – 0.25 | 0.050 |
| Condition [POST] × time | 0.06 | -0.12 – 0.23 | 0.511 |
| Condition [PRO] × time | -0.02 | -0.19 – 0.16 | 0.845 |
| **Random Effects** | | | |
| σ^2^ | 1308.79 | | |
| τ_00_ _ID_ | 2597.13 | | |
| N _ID_ | 16 | | |
| Observations | 240 | | |
| Marginal R^2^ / Conditional R^2^ | 0.081 / NA | | |

|  | **Methionine** | | |
| --- | --- | --- | --- |
| *Predictors* | *Estimates* | *CI* | *p* |
| (Intercept) | 29.92 | 27.03 – 32.82 | **<0.001** |
| Condition [POST] | -1.42 | -3.66 – 0.82 | 0.214 |
| Condition [PRO] | -1.44 | -3.68 – 0.80 | 0.206 |
| time | -0.02 | -0.04 – -0.00 | **0.013** |
| Condition [POST] × time | 0.01 | -0.01 – 0.04 | 0.194 |
| Condition [PRO] × time | 0.01 | -0.01 – 0.03 | 0.412 |
| **Random Effects** | | | |
| σ^2^ | 21.06 | | |
| τ_00_ _ID_ | 24.19 | | |
| N _ID_ | 16 | | |
| Observations | 240 | | |
| Marginal R^2^ / Conditional R^2^ | 0.039 / NA | | |

|  | **Ornithine** | | |
| --- | --- | --- | --- |
| *Predictors* | *Estimates* | *CI* | *p* |
| (Intercept) | 51.33 | 44.62 – 58.03 | **<0.001** |
| Condition [POST] | -2.98 | -7.36 – 1.40 | 0.181 |
| Condition [PRO] | 0.80 | -3.58 – 5.17 | 0.721 |
| time | 0.08 | 0.05 – 0.11 | **<0.001** |
| Condition [POST] × time | 0.03 | -0.01 – 0.08 | 0.129 |
| Condition [PRO] × time | -0.01 | -0.05 – 0.04 | 0.818 |
| **Random Effects** | | | |
| σ^2^ | 80.35 | | |
| τ_00_ _ID_ | 145.89 | | |
| N _ID_ | 16 | | |
| Observations | 240 | | |
| Marginal R^2^ / Conditional R^2^ | 0.321 / NA | | |

|  | **Phenylalanine** | | |
| --- | --- | --- | --- |
| *Predictors* | *Estimates* | *CI* | *p* |
| (Intercept) | 65.93 | 61.03 – 70.82 | **<0.001** |
| Condition [POST] | -3.58 | -7.46 – 0.29 | 0.070 |
| Condition [PRO] | -3.78 | -7.66 – 0.09 | 0.056 |
| time | 0.02 | -0.01 – 0.04 | 0.237 |
| Condition [POST] × time | 0.02 | -0.02 – 0.06 | 0.255 |
| Condition [PRO] × time | 0.00 | -0.03 – 0.04 | 0.800 |
| **Random Effects** | | | |
| σ^2^ | 62.96 | | |
| τ_00_ _ID_ | 67.57 | | |
| N _ID_ | 16 | | |
| Observations | 240 | | |
| Marginal R^2^ / Conditional R^2^ | 0.074 / NA | | |

|  | **Proline** | | |
| --- | --- | --- | --- |
| *Predictors* | *Estimates* | *CI* | *p* |
| (Intercept) | 187.44 | 160.35 – 214.52 | **<0.001** |
| Condition [POST] | -5.67 | -19.61 – 8.28 | 0.424 |
| Condition [PRO] | -4.17 | -18.12 – 9.77 | 0.556 |
| time | -0.05 | -0.15 – 0.04 | 0.290 |
| Condition [POST] × time | 0.09 | -0.05 – 0.22 | 0.220 |
| Condition [PRO] × time | 0.02 | -0.12 – 0.16 | 0.760 |
| **Random Effects** | | | |
| σ^2^ | 815.24 | | |
| τ_00_ _ID_ | 2620.57 | | |
| N _ID_ | 16 | | |
| Observations | 240 | | |
| Marginal R^2^ / Conditional R^2^ | 0.011 / NA | | |

|  | **Serine** | | |
| --- | --- | --- | --- |
| *Predictors* | *Estimates* | *CI* | *p* |
| (Intercept) | 92.41 | 82.27 – 102.55 | **<0.001** |
| Condition [POST] | -3.49 | -10.46 – 3.47 | 0.324 |
| Condition [PRO] | 2.73 | -4.24 – 9.70 | 0.441 |
| time | -0.00 | -0.05 – 0.04 | 0.883 |
| Condition [POST] × time | 0.06 | -0.01 – 0.13 | 0.086 |
| Condition [PRO] × time | 0.02 | -0.04 – 0.09 | 0.485 |
| **Random Effects** | | | |
| σ^2^ | 203.44 | | |
| τ_00_ _ID_ | 323.61 | | |
| N _ID_ | 16 | | |
| Observations | 240 | | |
| Marginal R^2^ / Conditional R^2^ | 0.042 / NA | | |

|  | **Threonine** | | |
| --- | --- | --- | --- |
| *Predictors* | *Estimates* | *CI* | *p* |
| (Intercept) | 122.61 | 107.72 – 137.50 | **<0.001** |
| Condition [POST] | -0.12 | -9.15 – 8.91 | 0.979 |
| Condition [PRO] | 1.81 | -7.22 – 10.84 | 0.693 |
| time | 0.00 | -0.06 – 0.06 | 0.973 |
| Condition [POST] × time | 0.05 | -0.03 – 0.14 | 0.232 |
| Condition [PRO] × time | 0.02 | -0.07 – 0.11 | 0.654 |
| **Random Effects** | | | |
| σ^2^ | 341.66 | | |
| τ_00_ _ID_ | 745.02 | | |
| N _ID_ | 16 | | |
| Observations | 240 | | |
| Marginal R^2^ / Conditional R^2^ | 0.023 / NA | | |

|  | **TotalAA** | | |
| --- | --- | --- | --- |
| *Predictors* | *Estimates* | *CI* | *p* |
| (Intercept) | 2840.59 | 2607.26 – 3073.92 | **<0.001** |
| Condition [POST] | -86.03 | -238.55 – 66.50 | 0.268 |
| Condition [PRO] | 3.28 | -149.25 – 155.80 | 0.966 |
| time | 0.34 | -0.72 – 1.41 | 0.525 |
| Condition [POST] × time | 1.19 | -0.32 – 2.69 | 0.121 |
| Condition [PRO] × time | 0.17 | -1.33 – 1.68 | 0.821 |
| **Random Effects** | | | |
| σ^2^ | 97512.57 | | |
| τ_00_ _ID_ | 176342.94 | | |
| ICC | 0.64 | | |
| N _ID_ | 16 | | |
| Observations | 240 | | |
| Marginal R^2^ / Conditional R^2^ | 0.014 / 0.649 | | |

|  | **Tryptophan** | | |
| --- | --- | --- | --- |
| *Predictors* | *Estimates* | *CI* | *p* |
| (Intercept) | 60.03 | 54.65 – 65.41 | **<0.001** |
| Condition [POST] | -0.42 | -4.68 – 3.84 | 0.846 |
| Condition [PRO] | 0.13 | -4.14 – 4.39 | 0.954 |
| time | -0.02 | -0.05 – 0.01 | 0.197 |
| Condition [POST] × time | 0.02 | -0.02 – 0.06 | 0.317 |
| Condition [PRO] × time | -0.01 | -0.05 – 0.04 | 0.757 |
| **Random Effects** | | | |
| σ^2^ | 76.16 | | |
| τ_00_ _ID_ | 81.63 | | |
| N _ID_ | 16 | | |
| Observations | 240 | | |
| Marginal R^2^ / Conditional R^2^ | 0.025 / NA | | |

|  | **Tyrosine** | | |
| --- | --- | --- | --- |
| *Predictors* | *Estimates* | *CI* | *p* |
| (Intercept) | 71.93 | 64.19 – 79.66 | **<0.001** |
| Condition [POST] | -3.91 | -10.30 – 2.47 | 0.228 |
| Condition [PRO] | -3.82 | -10.20 – 2.57 | 0.240 |
| time | 0.01 | -0.04 – 0.05 | 0.697 |
| Condition [POST] × time | 0.02 | -0.04 – 0.08 | 0.496 |
| Condition [PRO] × time | 0.02 | -0.05 – 0.08 | 0.606 |
| **Random Effects** | | | |
| σ^2^ | 170.95 | | |
| τ_00_ _ID_ | 162.44 | | |
| N _ID_ | 16 | | |
| Observations | 240 | | |
| Marginal R^2^ / Conditional R^2^ | 0.021 / NA | | |

|  | **Valine** | | |
| --- | --- | --- | --- |
| *Predictors* | *Estimates* | *CI* | *p* |
| (Intercept) | 253.67 | 226.72 – 280.62 | **<0.001** |
| Condition [POST] | -10.55 | -26.17 – 5.06 | 0.184 |
| Condition [PRO] | 0.71 | -14.91 – 16.33 | 0.929 |
| time | 0.12 | 0.01 – 0.23 | **0.032** |
| Condition [POST] × time | 0.05 | -0.11 – 0.20 | 0.547 |
| Condition [PRO] × time | -0.01 | -0.17 – 0.14 | 0.883 |
| **Random Effects** | | | |
| σ^2^ | 1022.14 | | |
| τ_00_ _ID_ | 2488.48 | | |
| N _ID_ | 16 | | |
| Observations | 240 | | |
| Marginal R^2^ / Conditional R^2^ | 0.077 / NA | | |
